# Workplace health promotion and safety in state and territorial health departments in the United States: a national mixed-methods study of activity, capacity, and growth opportunities

**DOI:** 10.1186/s12889-019-6575-x

**Published:** 2019-03-12

**Authors:** Laura A. Linnan, Maija S. Leff, Marisa C. Martini, AnnMarie L. Walton, Sherry Baron, Peggy A. Hannon, Jean Abraham, Melanie Studer

**Affiliations:** 1 Center for Health Promotion and Disease Prevention, University of North Carolina at Chapel Hill, 1700 M.L.K. Jr Blvd, CB#7426, Chapel Hill, NC 27514 USA; 2 Department of Health Behavior, Gillings School of Global Public Health, University of North Carolina at Chapel Hill, 135 Dauer Drive, CB# 7440, Chapel Hill, NC 27599 USA; 3 Health Systems and Analytics Division, School of Nursing, Duke University, 307 Trent Drive, Durham, NC 27710 USA; 4 Barry Commoner Center for Health and the Environment, Queens College, 65-30 Kissena Blvd, Flushing, NY 11367 USA; 5 Health Promotion Research Center, Department of Health Services, School of Public Health, University of Washington, 1107 NE 45th St., Suite 400, Box 354804, Seattle, WA 98105 USA; 6 Division of Health Policy and Management, School of Public Health, University of Minnesota, 420 Delaware St. SE, MMC 729, Minneapolis, MN 55455 USA; 7 Department of Health Policy and Management, Gillings School of Global Public Health, University of North Carolina at Chapel Hill, 135 Dauer Drive, CB #7411, Chapel Hill, NC 27599 USA

**Keywords:** State government, State health departments, Occupational health, One health, Health promotion, Centers for Disease Control and Prevention, National Institute for Occupational Safety and Health, Public health systems research

## Abstract

**Background:**

State and Territorial Health Departments (SHDs) have a unique role in protecting and promoting workers’ health. This mixed-methods study presents the first systematic investigation of SHDs’ activities and capacity in both Occupational Safety and Health (OSH) and Workplace Health Promotion (WHP) in the United States (US).

**Methods:**

National survey of OSH and WHP practitioners from each of 56 SHDs, followed by in-depth interviews with a subset of survey respondents. We calculated descriptive statistics for survey variables and conducted conventional content analysis of interviews.

**Results:**

Seventy percent (*n* = 39) of OSH and 71% (*n* = 40) of WHP contacts responded to the survey. Twenty-seven (*n* = 14 OSH, *n* = 13 WHP) participated in follow-up interviews. Despite limited funding, staffing, or organizational support, SHDs reported a wide array of activities. We assessed OSH and WHP surveillance activities, support that SHDs provided to employers to implement OSH and WHP interventions (implementation support), OSH and WHP services provided directly to workers, OSH follow-back investigations, and OSH standard and policy development. Each of the categories we asked about (excluding OSH standard and policy development) were performed by more than half of responding SHDs. Surveillance was the area of greatest OSH activity, while implementation support was the area of greatest WHP activity. Respondents characterized their overall capacity as low. Thirty percent (*n* = 9) of WHP and 19% (*n* = 6) of OSH respondents reported no funds at all for OSH/WHP work, and both groups reported a median 1.0 FTEs working on OSH/WHP at the SHD. Organizational support for OSH and WHP was characterized as “low” to “moderate”.

To increase SHDs’ capacity for OSH and WHP, interview respondents recommended that OSH and WHP approaches be better integrated into other public health initiatives (e.g., infectious disease prevention), and that federal funding for OSH and WHP increase. They also discussed specific recommendations for improving the accessibility and utility of existing funding mechanisms, and the educational resources they desired from the CDC.

**Conclusions:**

Results revealed current activities and specific strategies for increasing capacity of SHDs to promote the safety and health of workers and workplaces – an important public health setting for reducing acute injury and chronic disease.

**Electronic supplementary material:**

The online version of this article (10.1186/s12889-019-6575-x) contains supplementary material, which is available to authorized users.

## Background

State and Territorial Health Departments (SHDs) are tasked with ensuring the public’s health, including the health of workers. They are unique among organizations that endeavor to protect and promote workers’ health in the US. For example, when conducting occupational health surveillance, SHDs have the legal authority to mandate reporting of occupational injuries and illnesses by physicians (e.g. occupational asthma cases), as well as by laboratories (e.g. adult blood lead levels); a capability that provides worker health data not available through other agencies [1]. They also have the ability to create new occupational health data sources, by adding occupational health components to state-based health-related surveillance, such as the Behavioral Risk Factor Surveillance System [2]. SHDs can then use these data in collaborations with other government agencies to implement new state or local occupational safety and health (OSH) regulations [1]. SHD staff also have chronic disease expertise they can leverage to work with employers (directly or via local health departments) to offer workplace health promotion (WHP) programs, policies, and environmental supports. Furthermore, given their presence in all states, SHDs can advance multi-state initiatives by partnering with professional organizations such as the Association of State and Territorial Health Officials (ASTHO) or the Council of State and Territorial Epidemiologists (CSTE). Eleven states, for example, have worked with ASTHO and the Centers for Disease Control and Prevention (CDC) to reduce sodium intake among state employees by changing government food procurement policies [3]. Eight states (and the New York City Department of Health) participated in a surveillance initiative that tracked pesticide poisoning cases. This initiative identified illnesses associated with the use of pesticide-releasing foggers; working from these findings, the US Environmental Protection Agency (EPA) determined that labels on these products needed to be changed to improve user understanding of their risks and safe use [4]. Thus, SHDs have unique influence, reach and potential leverage for positively influencing the health and safety of the public. Their efforts are critical given that in 2015, an average of 13 workers died from work-related injuries each day in the US [5], and over 3.5 million non-fatal work-related illnesses and injuries were reported by US employers – approximately 9600 per day [6].

Despite the multiple roles that SHDs may play in protecting and promoting workers’ health, there has been no national assessment that examines the full range of activities that SHDs actually provide both in occupational safety and health (OSH) and workplace health promotion (WHP), as well as the interface between those activities within health departments. Assessing both fields jointly is particularly important as the Total Worker Health® approach gains traction [7]. The Total Worker Health® initiative at the National Institute for Occupational Safety and Health (NIOSH) supports research that explores the integration of occupational health protection efforts with interventions aimed at improving worker health and well-being. This approach acknowledges the importance of a holistic spectrum of factors – physical, organizational, and psychosocial – that present potential risks to employee safety and health. The purpose of this mixed methods study is to document the current level of OSH and WHP activity at SHDs, their capacity to engage in such activities, and to use these results to recommend how SHDs’ capacity for OSH and WHP activities might be increased.

## Methods

### Description of study design

**Fig. 1:**
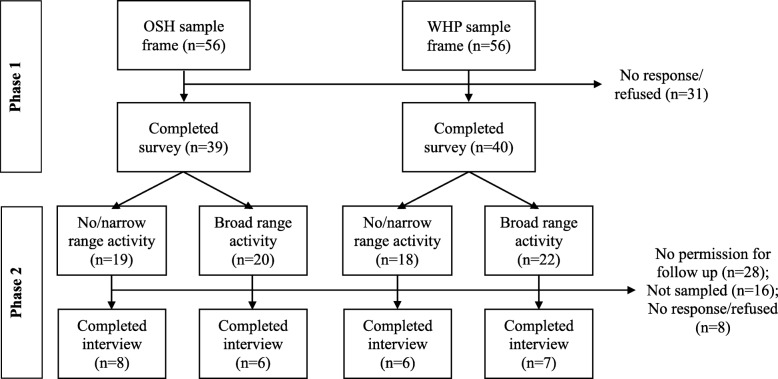
Study Design

We applied a two-staged “sequential embedded” mixed methods design (an adaptation of Creswell’s “concurrent embedded” design) [8]. In the first stage, we conducted a national, web-based survey of state and territorial health departments assessing their OSH and WHP activities, their current capacity for OSH and WHP activities, ideas for increasing OSH and WHP capacity in SHDs, and collaborations between OSH and WHP staff (the final topic is reported in a separate manuscript currently in preparation, not here). For stage two, we conducted follow-up, in-depth interviews with a sub-sample of the survey respondents. We used survey results to categorize SHDs by activity level and purposively recruited interview respondents from SHDs with both broad and narrow ranges of OSH and WHP activity to assess potential differences across this stratification variable. See Fig. 1 for a diagram of the study design.

### Sample & Procedures

#### Survey

We obtained the initial list of potential OSH survey contacts from NIOSH’s “State Health Department Occupational Safety and Health Contacts” directory [9]. To identify WHP contacts, we reviewed health department websites and the National Association of Chronic Disease Directors’ membership directory [10]. We included all 50 states and 5 inhabited territories (American Samoa, Guam, Northern Mariana Islands, Puerto Rico, and US Virgin Islands) as well as the District of Columbia. One author (MM) called each of the 112 (56 OSH and 56 WHP) contacts to confirm they were the “person with the best knowledge of [OSH or WHP, respectively] activities being performed by your Department.” If the contact was not the most knowledgeable person, we asked for a referral. In two states, the individual we confirmed as most knowledgeable about OSH was also confirmed as most knowledgeable about WHP, giving a final list of 110 non-duplicative contacts. All contacts received a pre-survey announcement via email, followed by a link to the survey. We sent two additional emails and made two follow-up calls to non-responders. Survey data were collected April through June 2016.

#### Interviews

We stratified survey respondents into four groups, based on their specialty (OSH vs. WHP) and the level of activity they reported on the survey. These were: broad OSH activity, narrow/no OSH activity, broad WHP activity, and narrow/no WHP activity. We asked all survey respondents if they would be willing to participate in a follow-up interview. From those who gave their permission to be contacted for a follow-up interview (*n* = 24 OSH and *n* = 27 WHP, 62 and 68% of respondents, respectively), we recruited at least six from each of the four activity levels. To reach six respondents for each activity level, we first recruited all OSH and WHP respondents from states where both had given their permission to be contacted for follow-up interviews (n = 24). If respondents declined or did not respond, we then pulled a stratified random sample from the remaining eligible contacts until the quota for each of the four stratum was reached (total non-response and declined: *n* = 8). Interviews were conducted September through November 2016.

### Data Collection Instruments & Measures

#### Survey

We developed and pilot tested the survey with experts in both OSH and WHP, including specialists from the Workplace Health Research Network (WHRN) [11], and three SHD representatives. Key items are described below and displayed in Table 1. The complete survey is included as supplemental files (Additional file 1: OSH version; Additional file 2: WHP version).

**Table 1:** Key Survey Items (complete survey available in additional files)

Activities	Question: The following activities are performed (check all that apply): by your Health Department, independently; in partnership with another organization(s); by another organization(s), on behalf of your department (*WHP*)/ by a “bona-fide agent,” acting on behalf of your Health Department (*OSH*); your Health Department has not performed this activity in the last 12 months	
	Surveillance	*WHP*: Monitoring of Healthy People 2020 worksite-related objectives; Monitoring of other workplace health promotion activities in the state, beyond the Healthy People 2020 objectives *Scoring: “high” if both activities, “some” if one, “none” if neither*
		*OSH*: Compile, analyze, and interpret Occupational Health Indicators (If “yes”: What proportion of these 22 indicators are compiled, analyzed, and interpreted in your state?); Workers’ Compensation surveillance, beyond the collection of Workers’ Compensation-related OHIs; Surveillance of occupational lead levels in adults and submission of data to the NIOSH Adult Blood Lead Epidemiology and Surveillance program; Monitoring data from the National Surveillance System for Pneumoconiosis Mortality; Monitoring of indicators for the Healthy People 2020 occupational safety and health objectives; Targeted surveillance for Fatality Assessment, Control, and Evaluation; Targeted surveillance of occupational respiratory disease; Targeted surveillance of pesticide illness and injury; Targeted surveillance of musculoskeletal disorders; Targeted surveillance of “target worker populations” (e.g., youth, older age workers, immigrant workers, temporary workers, and workers in high-risk industries/occupations); Presentation of OSH surveillance data to relevant staff (occupational health professionals and health care providers) at workplaces within your state. *Scoring: “high” if 4 to 9 points, “some” if 1 to 3 points, “none” if no points. 0 to 6 points assigned based on proportion of the 22 Occupational Health Indicators compiled, analyzed, and interpreted; 0 to 3 points assigned based on proportion of 10 additional surveillance activities completed*
	Implementation support to employers	*WHP*: Providing …educational materials, resources and/or toolkits to employers; …training to employers, such as training sessions and/or webinars designed to introduce a group of employers to a workplace health promotion topic; …technical assistance to employers, such as individualized, on-demand consultations and/or phone-based support; …employers with assistance in monitoring the quality of their workplace health promotion programs, for example through providing organizational audits, health assessments, and/or other evaluation resources.
		*OSH*: Development and dissemination of educational materials to employers; Providing …training and education programs to employers; …technical assistance to employers, upon request; …employers with assistance in monitoring the quality of their occupational safety and health programs, for example through providing organizational audits or other evaluation resources. *Scoring for both WHP and OSH: four, three, two, one, or no implementation support activities provided.*
	Direct services to workers	*WHP*: Delivering health promotion programs directly to workers across your state, such as flu shots or blood pressure screening programs.
		*OSH*: Providing educational materials, training, and/or technical assistance to workers directly. *Scoring for both WHP and OSH: “providing” or “not providing”.*
	Follow-back investigation	*OSH*: Worksite follow-back investigations; Referrals of employers/worksites to OSHA or other agencies for follow-back investigations. *Scoring: “both”, “either”, or “neither”*
	Standard and policy development	*OSH*: Standard and policy development. *Scoring: “active” or “inactive”*
Capacity	Financial resources	*OSH and WHP*: Health Department’s total funding for OSH (or WHP) activities during the past 12 months, including federal, state, and other funds. *Free response.*
	Human resources	*OSH and WHP*: Total number of FTEs performing OSH (or WHP) activities in the Health Department during the past 12 months. Include all those employed by the state, all those working at the state-level who are either federal assignees or contract employees, and state employees assigned to work in a regional office. *Free response.*
	Competency	*WHP*: Health Department’s level of competency (knowledge and skills) to perform the following workplace health promotion activities: Workplace health promotion surveillance activities; Implementation support activities (providing support to employers with design and implementation of workplace health promotion initiatives); Direct service activities (delivering workplace health promotion services directly to employees across your state). *“Minimal or none”, “Basic”, “Intermediate”, “Advanced”,* or *“Expert”*
		*OSH*: Health Department’s level of competency (knowledge & skills) to perform the following occupational safety and health activities: Occupational safety and health surveillance activities; Follow-up intervention and prevention activities; Direct service activities (delivering occupational safety & health services directly to employees). *“Minimal or none”, “Basic”, “Intermediate”, “Advanced”,* or *“Expert”*
	Organizational support	*OSH and WHP*: Health Department’s commitment to OSH (or WHP)? *“Not at all”, “Slightly”, “Moderately”, “Very”, “Extremely*”. What level of priority does your Health Department assign to OSH (or WHP) efforts, in comparison to other efforts your Health Department is involved in? *“Not a priority”, “Low level”, “Moderate level”, “High level”, “Very high level”*
	Overall capacity	*OSH and WHP*: Health Department’s overall capacity to support workplace health promotion among employers in the state? *“No capacity”, “Minimal”, “Some”, “Moderate”, “Substantial”*

##### Activity items

We assessed three types of activity for both OSH and WHP: surveillance, implementation support to employers, and direct services to workers. Two types were assessed for OSH respondents only: standard and policy development and follow-back investigation. Respondents reported on activities from the past 12 months and indicated if their SHD did these: 1) independently, 2) in partnership with other organizations, 3) through a contracted organization [*WHP only*]/through a bona-fide agent^1^ [*OSH only*], or 4) not at all. Respondents could select more than one way of doing an activity. We considered an SHD “engaged” in the activity if respondents reported that it was done in any form. *Summary Activity Score:* We combined the subscales to create a summary OSH activity score (summing all 5 subscales) and a summary WHP activity score (summing 3 subscales). We equally weighted subscales before summing and applied a median split on the summary activity score to identify SHDs with a “broad range” of OSH or WHP activities, vs. a “narrow range” or no OSH or WHP activities. A median split was chosen to make comparisons between more and less active SHDs within the range of activities SHDs actually conducted. Additionally, while established guidelines for OSH activities in SHDs exist [12], there were none for WHP activities at the time of this study, precluding grouping by an existing standard for both disciplines.

##### Capacity items

We assessed five areas identified [13–17] as critical components of organizational capacity: Human Resources, Financial Resources, Competency, Partnerships, and Organizational Commitment. See Table 1 for detail on items and response scales.

After assessing existing capacity, we asked about the type of 1) support or training SHDs would need to improve their competency (knowledge and skills) to perform OSH/WHP; and 2) assistance SHDs would need to increase capacity to support OSH/WHP (both free text response).

#### Interview guide

The interview guide was developed with input from both OSH and WHP experts from the WHRN, as well as practitioners from state/county health departments and NIOSH representatives. In-depth questions covered funding, staffing, leadership/organizational support, training resources, resources needed to expand activities, partnerships and context. Selected items are described in Table 2. The complete interview guide is available as a supplemental file (Additional file 3).

**Table 2:** Sample Interview Questions (complete interview guide available in additional files)

What do you think your department would need the most right now if it wanted to expand its efforts in OSH (or WHP)?	
What do you think it is important for us to know when it comes to funding for OSH (or WHP) programming?	
In your opinion, what might your health department’s ideal staffing be to do OSH (or WHP) work in this state?	
What could your health department leadership do to better support OSH (or WHP) in your state? Can you think of any ways the CDC could help health department leadership become more supportive of OSH (or WHP)?	
In the survey, we asked about helpful resources that your health department uses to conduct OSH (or WHP) work. You replied [*response*] were the most helpful resources. Pick the one you consider to be the most helpful – In what way did you use it? What characteristics made it so helpful? Is there anything you would change?	

### Analysis

We calculated descriptive statistics for all survey variables and summary activity scores using SAS. We used conventional content analysis [18] to analyze the interview data. The project manager (ML) created the initial codebook integrating both deductive and inductive codes after line-by-line coding of two transcripts. Deductive codes came from Meyer et al.’s definition of organizational capacity [16]. Three members of the coding team (ML, MM, and AW) independently applied the codes to each of two transcripts, then met to resolve coding discrepancies and revise the codebook as needed. The team agreed on code application in the first two transcripts, and split the remaining 25 for independent coding. We refined the codebook by group consensus until the final transcript was coded. We summarized the coded data for each interview and entered it into matrices organized by topic (e.g., all codes related to leadership support included in one matrix, all codes related to partnerships in another). A single team member independently populated each matrix and adjusted code applications in the transcripts if needed. After populating these matrices, team members wrote analytical memos summarizing themes and relationships in the matrices. Throughout, we compared findings for respondents from SHDs with broad OSH/WHP activity against those for respondents from narrow/no OSH/WHP activity SHDs. We edited the analytical memos through team discussion which then served as the interview results.

This manuscript reports on the survey and interview data related to OSH/WHP activities, OSH/WHP capacity, and respondents’ ideas for increasing OSH/WHP capacity. Study findings about the partnerships between OSH/WHP programs and external organizations and about Total Worker Health® efforts in SHDs are in preparation.

## Results

### Sample description

Seventy percent (39/56) of OSH survey contacts responded to the survey, 7% (4/56) refused, and 23% (13/56) did not respond. Seventy-one percent (40/56) of WHP survey contacts responded to the survey, 2% (1/56) refused, and 27% (15/56) did not respond.

OSH survey respondents most commonly worked in divisions of surveillance/epidemiology, environmental health, occupational health, and health promotion/chronic disease prevention. Two were from state agencies other than the SHD, and two were bona fide agents for their SHD. WHP survey respondents most commonly worked in divisions of health promotion/chronic disease prevention, nutrition, physical activity, and family or community health. Typically, respondents in both groups held leadership positions (e.g., bureau director, division chief), worked in program managerial positions (WHP), or worked as the State Epidemiologist or other epidemiologist (OSH).

As intended based on quota sampling, WHP interview respondents were from SHDs with either broad (*n* = 7) or narrow range/no (*n* = 6) WHP activities; OSH interview respondents were from SHDs with either broad (n = 6) or narrow range/no (*n* = 8) OSH activities. OSH respondents were both from states that were funded through the NIOSH State Occupational Health and Safety Surveillance Program (*n* = 9),^2^ and unfunded states (*n* = 5).

### Current workplace health and safety activities

Overall, 92% (*n* = 36) of OSH survey respondents reported that their SHD engaged in at least one OSH surveillance activity, and 67% (*n* = 26) reported a high number of surveillance activities (SHDs scored as “high” on OSH surveillance activities typically collected 15+ OHIs and conducted an average of 5 other surveillance activities) (Table 3). The most commonly reported OSH activities were: surveillance of occupational lead levels in adults and submitting data to the NIOSH Adult Blood Lead Epidemiology and Surveillance (ABLES) program (85%, *n* = 33); compiling, analyzing, and interpreting the Occupational Health Indicators (OHIs) (72%, *n* = 28); presenting surveillance data to workplaces within their state (62%, *n* = 24); Workers’ Compensation surveillance (beyond Workers’ Compensation-related OHIs) (49%, *n* = 19); and surveillance of target worker populations (e.g., youth workers, immigrant workers) (49%, *n* = 18) (see Additional file 4: Occupational Safety and Health (OSH) Surveillance Activities for the prevalence of all assessed activities). Among WHP survey respondents, 71% (*n* = 27) monitored Healthy People 2020 worksite-related objectives, and 65% (*n* = 23) monitored other WHP activities in the state such as indicators from the CDC Worksite Health ScoreCard [19], state physical activity or nutrition plans, or state worksite wellness surveys. Eighty-two percent (*n* = 32) conducted at least one of these WHP surveillance activities, and 46% (n = 18) collected data both on Healthy People 2020 indicators and on other WHP activity indicators (categorized as “high” activity).

**Table 3:** State Health Department Activities in Occupational Safety and Health (OSH) and Workplace Health Promotion (WHP)

	OSH survey respondents (*n*=39)	WHP survey respondents (*n*=40)
Level of surveillance activity SHDs engage in		
None	8% (3)	18% (7)
Some	26% (10)	36% (14)
High	67% (26)	46% (18)
Percent who provided each of the following types of implementation support to employers		
Tools & educational materials to employers	68% (25)	90% (36)
Training to employers	41% (15)	78% (31)
Technical assistance to employers	59% (22)	79% (31)
Assist employers in monitoring program quality	18% (6)	64% (25)
How many types of implementation support did SHDs provide to employers?		
None	28% (11)	10% (4)
1	21% (8)	8% (3)
2	13% (5)	8% (3)
3	26% (10)	15% (6)
All 4	13% (5)	60% (24)
Percent (Yes) who provided direct services to workers	61% (23)	51% (20)
Percent who engaged in follow-back investigation activities (OSH only)		
None	31% (12)	…
EITHER conduct investigations OR refer to other agencies for investigations	28% (11)	…
BOTH conduct investigations AND refer to other agencies for investigations	41% (16)	…
Percent (Yes) who engaged in standard and policy development? (OSH only)	39% (15)	…

Seventy-two percent (*n* = 28) of OSH survey respondents reported their SHD provided at least one type of OSH implementation support to employers, but only 13% (*n* = 5) reported providing all four (Table 3). SHDs were considerably less likely to provide OSH quality assurance/improvement (18%, *n* = 6) to employers than any other type of OSH implementation support. Among WHP survey respondents, 90% (*n* = 36) reported that their SHD provided at least one type of WHP implementation support, while 60% (*n* = 24) reported that their department provided all four. All WHP implementation supports were provided by a majority of SHDs. Reach varied by activity, and was highest for tools and educational materials among both OSH and WHP programs, with 24%, (*n* = 9) of OSH and 65% (*n* = 26) of WHP survey respondents reporting that their SHD reached > 25 employers with tools and educational materials in the past year (see Additional file 5 for detailed breakdown of how many employers were reached by OSH and WHP implementation supports).

Providing OSH/WHP services directly to workers was common, with more than half of both OSH and WHP survey respondents saying their SHD engaged in this activity (see Table 3). OSH standard and policy development was the least common activity of all, with 39% (*n* = 15) of OSH survey respondents reporting their SHD engaged in this.

### Current capacity and respondents’ recommended strategies for increasing capacity

#### Funding and staffing

OSH survey respondents reported their SHD had a median budget of $150,000 for OSH activities over the past 12 months. Nineteen percent (n = 6) reported no OSH funding at all. WHP survey respondents reported having a median budget of $57,500 for WHP activities. Thirty percent (n = 9) reported no funds for WHP at all. Significant funding for these programs was rare, with only 13% (*n* = 4) of OSH and 3% (n = 1) of WHP respondents reporting more than $500,000 in funding annually. Both OSH and WHP survey respondents reported a median of 1.0 FTE working on activities over the last 12 months. The interquartile range for FTEs working on OSH activities was 0.3 FTEs – 4.5 FTEs. For FTEs working on WHP activities it was 0.1 FTEs – 1.5 FTEs (Table 4).

**Table 4:** State Health Departments' Capacity for Occupational Safety and Health (OSH) and Workplace Health Promotion (WHP)

	OSH survey respondents (*n*=39)	WHP survey respondents (*n*=40)
Total funding for OSH/WHP activities over the past 12 months		
Median	$150,000	$57,500
$0	19% (6)	30% (9)
Up to $50 K	13% (4)	17% (5)
$51 K - $150 K	26% (8)	30% (9)
$151 K - $500 K	29% (9)	20% (6)
More than $500 K	13% (4)	3% (1)
Total FTEs conducting OSH/WHP activities over the past 12 months		
Median	1 FTE	1 FTE
Interquartile range	0.3 FTE – 4.5 FTE	0.1 FTE – 1.5 FTE
Perception of SHDs’ competency to perform the following:		
Surveillance activities		
Advanced-Expert	58% (21)	44% (17)
Basic-Intermediate	33% (12)	49% (19)
Minimal or None	8% (3)	8% (3)
Implementation support/Follow-up intervention and prevention activities*		
Advanced-Expert	31% (11)	56% (22)
Basic-Intermediate	53% (19)	36% (14)
Minimal or None	17% (6)	8% (3)
Direct service activities		
Advanced-Expert	22% (8)	35% (13)
Basic-Intermediate	36% (13)	43% (16)
Minimal or None	42% (15)	22% (8)
Organizational and leadership support		
SHD’s commitment to OSH/WHP		
Very-extremely	30% (11)	44% (17)
Slightly-moderately	56% (20)	56% (22)
Not at all	14% (5)	0% (0)
Level of priority SHD assigns to OSH/WHP		
High-very high	13% (5)	21% (8)
Low-moderate	66% (25)	74% (28)
Not a priority	21% (8)	5% (2)
Overall capacity to support employers with OSH/WHP in the state (self-reported)		
Moderate-substantial	21% (8)	33% (13)
Minimal-some	68% (26)	62% (24)
None	11% (4)	5% (2)

*WHP respondents were asked to rate their SHD’s competency for “Implementation support activities”, while OSH respondents for “Follow-up intervention and prevention” activities. See methods for definitions

During interviews both OSH and WHP respondents suggested that funding could be increased by reducing the burden of federal grant application and management processes, and requested greater stability in funding sources from year to year (see Table 5). OSH interview respondents additionally requested that states be given flexibility in how they use available funds, and requested that the NIOSH State Occupational Health and Safety Surveillance Program applications focus more on public health practice rather than research. The latter request was only seen among OSH respondents from SHDs with a narrow range of OSH activities.

**Table 5:** Interview Respondents’ Recommendations for Improving Funding Mechanisms for Occupational Safety and Health (OSH) and Workplace Health Promotion (WHP)

Recommendation	Barrier addressed
OSH	
Reduce the number of requirements associated with the NIOSH State Occupational Health and Safety (OHS) Surveillance Program applications.	SHD leadership viewed application requirements as too time-consuming, prohibiting submission: “*…there are letters of support required. Well, because this is a brand new program and there’s such a long wait time on applying for it…our management is unwilling for me to go out and try and start. It wants you to actually start having a committee…and they don’t want us to do that…*” (OSH7)
Revise the NIOSH State OHS Surveillance Program application to focus on public health practice rather than research.	Funding surveillance through a research grant created administrative burden and limited the OSH practice activities SHDs could engage in: “…*the way the funding occurs is…it’s actually a research grant…And the fact that it’s a research grant significantly limits our ability to conduct certain activities…we have to consider ‘is this research activities, does it need to be approved by IRB?’ and the fact that it’s research typically says we’re trying to make generalizations about other populations, when a state-based health program should be aimed at trying to improve the population in our state*” (OSH6)
Give states greater flexibility in how grant funds are used.	Flexibility would give SHDs increased ability to respond to emerging priorities: “*Right now our staff almost entirely, each one, is tied to a specific funding source that dictates what they’re able to work on and that really doesn’t leave us with staffing that we can decide what they should work on*” (OSH4)
WHP	
Provide more resources within grants for administrative and grant management personnel.	This will give SHDs the grant management infrastructure that allows them to expand programming: “*So we’ve turned down at least 2 or 3 funding opportunities because we didn’t have the bandwidth to add the bureaucratic levels to deal with that and manage that funding. So, I think that building into funding opportunities ways to reach that would be really useful*” (WHP2)
Provide greater stability in funding sources from year to year.	Stable funding would ensure that fluctuations in funding wouldn’t undo the SHD’s capacity to expand or continue WHP activities that were previously supported: “*It’s hard when funding comes and goes, you know? It’s just like the latest greatest this year, but then next year it’s taken away. And so efforts come and efforts go, which I think is really sad*” (WHP5)

Both OSH and WHP interview respondents, irrespective of activity level, said that hiring more staff was necessary to expand activities, form partnerships, and collaborate with their respective OSH/WHP counterpart. Hiring additional staff (and retaining quality staff) was described as contingent on having additional funding. In particular, respondents sought funding for coordinating/program management staff: *“…there are no staff to deliver or coordinate [this work]…There’s always got to be someone on point, coordinating something of this magnitude … and if it’s an important issue, it needs resources, quite honestly.”* (WHP1) Some WHP interview respondents desired funding to be able to assign a staff person to each region in the state to recruit and provide technical assistance to employers implementing WHP programming.

#### Organizational and leadership support

Overall, organizational support for OSH and WHP activities was rated low to moderate among survey respondents. The majority of both OSH and WHP survey respondents said that their SHD was only “slightly” or “moderately” committed to OSH/WHP (OSH: 56%, *n* = 20; WHP: 56%, *n* = 22), and an even higher proportion reported that OSH/WHP was a “low” or “moderate level” priority for their SHD (OSH: 66%, *n* = 25; WHP: 74%, *n* = 28).

During interviews, both OSH and WHP respondents, regardless of activity level, identified two foundational changes that could increase leadership support for OSH/WHP. First, respondents proposed that OSH/WHP approaches needed to be fully integrated into the work of other programs at the SHD (see Table 6). Specifically, they suggested including OSH/WHP experts as part of multi-disciplinary teams within the department; this would bring critical worker health perspectives to other public health efforts. Another suggestion for integration, made by WHP interview respondents, was to use WHP programs to coordinate other chronic disease efforts at the worksite. Both OSH and WHP interview respondents emphasized that integration would give leadership opportunities to see how OSH/WHP practices could help the department achieve its other public health goals, building leadership interest in OSH/WHP. Additionally, integration would allow poorly-funded workplace programs to tap into partners’ funding streams. In states where there was no leadership support for starting an OSH/WHP program, interview respondents noted that integration would be the only way to access funding (see Table 6).

**Table 6:** Interview Respondents’ Recommendations for Building Organizational and Leadership Support for OSH and WHP

Recommendation 1: Integrate OSH/WHP approaches into other public health initiatives.	
Benefits of integration	For the Health Department • Including OSH/WHP expertise on teams brings a specialist perspective that improves program outcomes: “…*if you are trying to address infection control or Ebola…the people who know the most about personal protective equipment (PPE) are occupational health and safety people, and those are the people that need to be at the table when you’re talking about PPE requirements…so you need to have more of a team approach.*” (OSH1) • Using WHP to coordinate chronic disease efforts at the worksite fosters a more strategic approach and allows chronic disease programs to expand reach: “…*there must be some sort of worksite objective or goal that, the money that goes to the chronic disease program, can go to that. Because that’s where so much of their reach needs to be is in the worksite and they’re not taking advantage of it…or if they do work with the worksite, it’s very limited. Maybe just something on blood pressure or something on diabetes. So they miss the big picture piece where we go in there with the CDC Health ScoreCard and we do an assessment and then we figure out what does this worksite really need to focus on.”* (WHP3) For OSH/WHP programs, specifically • Integration boosts leadership awareness of the contributions of OSH/WHP to health department goals: “*You know, occupation is not listed over there as the agency priority. But we can say through occupational health surveillance…what kind of mother in what kind of occupation, what kind of industry is more likely to have a pre-term or very low-birthweight baby...So through this link…the agency says ‘Oh! Occupational surveillance can help the agency to achieve the agency’s goal…*’” (OSH2) • Integration provides access to other funding streams coming into the department (especially important when existing OSH/WHP capacity precludes applying for targeted funding): “…*since we’re not likely to start from scratch and establish [OSH] as a new program area…I think that CDC looking at ways that OSH can be incorporated into other programs…kind of more of a “one health” approach is probably gonna be more successful…for example there’s a lot of money that gets funneled into health care associated infections. And that’s a program we have here. So if there are tools and resources and funding towards expanding HIE surveillance programs to also include some aspects of occupational health and safety, I think that would be the most successful way to go*.” (OSH3)
Strategies for promoting integration	• CDC can fund integration by specifying that a certain amount of existing awards be applied to OSH/WHP activities, make collaboration with OSH/WHP a requirement to receive funding, or require integrated teams.
Recommendation 2: Recognize OSH/WHP as core disciplines in public health.	
Barriers to recognizing OSH/WHP as core public health	• NIOSH funding only half of states for OSH surveillance perpetuates view that OSH is optional/elective: *“There’s no consistent model for occupational safety and health in a public health department, that you can reliably expect to be there for every single state in the country…like half the states in the country don’t have anybody in the public health department!…it’s a real shame…that there is no consistent, core funding or expectation for occupational safety and health in public health.”* (OSH8) • OSH not viewed as public health’s responsibility because of other state agencies (e.g., Department of Labor) that work in this field: “*I’m asked all the time ‘What do you need more resources for in Occupational Health, doesn’t OSHA take care of that?’”* (OSH4)
Strategies for promoting OSH/WHP as core disciplines in public health	• NIOSH fund all SHDs for basic OSH surveillance: “*I think it should be essential funding, by the federal government…because there is no clear commitment from CDC, OSHA, or federal government…the locals don’t see it as a problem.”* (OSH5) • Add industry and occupation indicators to all major public health data sets (e.g., BRFSS) • CDC encourage inclusion of occupational and environmental objectives in State Healthy People objectives • Increased federal funding for WHP • CDC release more WHP-focused tools (e.g., CDC Worksite Health ScoreCard) and training: *“…we haven’t had that coming from CDC saying this is important and we’re going to have a conference...But I think those kind of things are what gets the SHD leaders and staff thinking ‘Oh so this matters too, and it’s a part of all that we do’”* (WHP3)

Both OSH and WHP interview respondents, however, indicated that their SHDs needed incentives from the CDC in order to integrate OSH/WHP into other public health programs. They recommend that the CDC fund integration, by requiring that “*other streams of funding that come to Health Departments carve out a component for worker health,*” (OSH4) or by making collaboration with OSH/WHP a requirement to receive funds. The CDC could also encourage integrated teams, telling departments “*… you need to have an occupational health and safety person as part of your infection control group, as part of your response group…*” (OSH1).

However, the low level of integration of OSH/WHP into other divisions was related to a larger issue: both OSH and WHP interview respondents said these fields were not seen as core disciplines in public health, and thus not prioritized by leadership. OSH interview respondent**s** thought that NIOSH’s practice of only funding a portion of states to do OSH surveillance perpetuated the perception that occupational health was an elective or auxiliary practice in public health. They also noted that some leaders in the SHD mistakenly thought that other worksite-focused agencies (such as OSHA) had assumed responsibility for all OSH work, thereby removing it from the purview of public health. Therefore, the second foundational change respondents sought was for SHD leadership to recognize OSH and WHP as core disciplines in public health. They recommended a variety of strategies to achieve this change. OSH interview respondents called on NIOSH to fund all states for OSH surveillance at a basic level. To help colleagues recognize employment as a core public health determinant, they recommended that industry and occupation indicators be added to all major public health data sets such as the Behavioral Risk Factor Surveillance Survey System (BRFSS). They also recommended that the CDC encourage inclusion of occupational and environmental objectives in State Healthy People objectives. WHP interview respondents called for increased funding and also requested that the CDC release more WHP-focused tools, such as the CDC Worksite Health ScoreCard and workplace health and safety conferences (see Table 6). Respondents anticipated that increased leadership support would have cascading positive effects on capacity, with greater access to funding (including state general funds), partnerships, and staff.

#### Competency for OSH and WHP

Survey respondents’ perception of their departments’ competency (knowledge and skills) to perform OSH/WHP activities varied across the different types of activity (see Table 4). OSH survey respondents were most likely to report a high level of competency (“advanced” or “expert”) for OSH surveillance (58%, *n* = 21), while WHP survey respondents were most likely to characterize their departments as “advanced” or “expert” in implementation support activities (56%, *n* = 22). Both groups of respondents were least likely to rate their departments as highly competent in direct services to workers (OSH: 22%, *n* = 8 and WHP: 35%, *n* = 13).

To build SHD staff competency (knowledge and skills), both OSH and WHP interview respondents recommended peer learning collectives, educational resources designed to train newcomers to the field, and accessible trainings. Peer learning collectives gave respondents access to examples of peers’ projects, where they could see the “*step by step process*” (WHP4) required for successful execution. Respondents pointed out that peer support was especially critical in a low resource setting: “*you know, when you don’t have staff or capacity, you don’t have time to reinvent the wheel, so we learn from our colleagues in other states*” (WHP5). Professional meetings such as the Southern States Occupational Network [20] meetings and CSTE’s Occupational Health Surveillance Subcommittee [21] meetings were given as current examples of valuable peer learning opportunities. Educational resources designed to train newcomers to the field were also valued as several respondents noted it was common to hire staff who had no background in workplace health. Interview respondents also sought accessible trainings, (e.g. online, low-cost, and/or open to community partners). The topics respondents desired varied. Two common themes were training on how to make the business case for WHP, and on specific occupational epidemiology practices. No consistent differences across activity strata were observed.

## Discussion

This was the first national survey of US State and Territorial Health Departments (SHDs) to assess current activities and capacity both for occupational safety and health (OSH) and workplace health promotion (WHP). A high response rate to the online survey combined with additional in-depth interviews with OSH and WHP respondents from departments with different ranges of activity yielded important information about activities and opportunities to build future capacity at these important public health institutions.

Given current federal funding priorities, we were not surprised to find that SHDs were most active in surveillance activities for OSH and program implementation support for WHP. The primary source of federal funds for OSH activities is surveillance-focused (NIOSH’s State Occupational Health and Safety Surveillance Program [22]). At the time of this survey, the primary federal funding for WHP activities came through two non-recurring State and Local Public Health Actions (1305 [23] and 1422 [24]) with the short-term goal of improving environments in worksites to promote healthy behaviors. This focus on the worksite environment likely encourages direct engagement with employers (i.e., implementation support), as they are gatekeepers for environmental changes at their worksites. Notably, having federal funds that encourage OSH and WHP programs to focus on different types of activity (e.g. surveillance, direct service, implementation support or follow-back investigations) can positively impact SHDs’ ability to engage in Total Worker Health® initiatives. As the CDC and SHDs develop models for Total Worker Health® that involve SHDs, it should build capacity and interest in these collaborations.

In addition to current activities, we also assessed the capacity SHDs had for OSH/WHP work across five key domains (Human Resources, Financial Resources, Competency (knowledge and skills), Partnerships, and Organizational Commitment). Although respondents reported low levels of capacity, we found that each of the activities we asked about (excluding OSH standard and policy development) were performed by more than half of the responding SHDs. Thus, our results indicate that despite low capacity, SHDs are conducting a variety of WHP and OSH activities. As emphasized by survey and interview respondents, we agree that expanding resources (funding and/or staffing) for these programs could increase the amount and range of activities, with great potential for positive impact on workers’ health.

Survey respondents reported relatively low or no funding for this work. As a consequence of poor funding, approximately one quarter of both OSH and WHP programs were operating with extremely limited staffing (≤0.3 FTE and ≤ 0.1 FTE, respectively), and half worked with 1 or fewer FTEs. This resource-scarce picture matches what we know from federal funding records. Despite the fact that more than 60% of adults work, CDC support for OSH and WHP initiatives in SHDs is considerably lower than that for many other aspects of SHD public health programs. For example, in FY2016, states and territories received a total of $96 million in grant dollars from the CDC for Occupational Safety and Health – or 2.7% of the CDC’s total grant funding to states and territories. In comparison, states and territories received $733 million for HIV/AIDS, Viral Hepatitis, STI, and TB Prevention, and $608 million for Public Health Preparedness and Response. Chronic Disease Prevention and Health Promotion was the CDC’s best funded grant area in 2016 ($758 million, or 21.5% of total 2016 funds) [25], but WHP projects were only an optional activity under one subset of chronic disease funding grants. Additionally, the majority of respondents perceived their SHD’s support for OSH or WHP to be at best, moderate. Considering these realities and perceptions, it is not surprising that the majority of both OSH and WHP respondents assessed their department’s overall capacity to support employers with OSH or WHP as “minimal” to “some”.

To increase funding for OSH and WHP activities by SHDs as a way to increase capacity, interview respondents had a number of specific and actionable recommendations, including: 1) reduce the number of requirements associated with applying for State OHS Surveillance funds, so as not to dissuade low-capacity states from applying; 2) refocus the State OHS Surveillance Program on public health practice, rather than research; 3) give states greater flexibility in how OSH grant funds are used, so that they can effectively respond to emerging concerns; 4) provide more resources within existing WHP grants for administrative/grant management personnel; and 5) provide greater stability in funding streams from year to year. While an overall increase in funding is most desirable, changes to the way current funds are implemented and allocated could also have positive outcomes for both OSH and WHP capacity to do work independently, and in a more integrated fashion as well.

While funding is necessary, it is not a sufficient condition for improving SHD capacity to do this work. According to respondents, leadership support for OSH/WHP activities is critically important and would be enhanced by integrating OSH/WHP activities into other public health initiatives. Our respondents echo earlier calls by OSH professionals in SHDs to integrate OSH with public health [1]. Davis and Souza [1], as well as Baron et al. [26] have presented case studies of health departments engaged in integrative work, which may serve as examples for other SHDs moving forward. Both California’s and Connecticut’s SHDs have trained food safety inspectors to conduct outreach about worker safety hazards in restaurants, integrating OSH services into the licensing functions of the SHD. The *Cleaning for Asthma-Safe Schools* project (also in California) protected the health of children, teachers and custodial staff by helping schools transition to cleaning supplies and practices that did not promote asthma. By removing dangerous cleaning supplies, an occupational hazard for custodians, the project was able to protect both workers’ and children’s health [27]. The Massachusetts SHD has conducted a number of integrated initiatives, including developing a multi-disciplinary team to address latex-related asthma (triggered by latex gloves) among workers and clients in healthcare, food service, emergency response, and child-care settings [1]. CDC funding that requires OSH/WHP staff to contribute their expertise to larger chronic disease/infectious disease/injury prevention programs simultaneously builds capacity for OSH and WHP programming, and, reduces silos within SHDs that act as barriers to integration. Therefore, we believe integration strategies like Total Worker Health® will become a valuable strategy for increasing capacity even in states with very low existing leadership support, as integration channels financial and human resources towards OSH/WHP activities.

Many interview respondents perceived that their SHD leadership did not prioritize OSH and WHP initiatives because worker health was not considered to be a core responsibility of public health departments. As a result, respondents indicated that they faced challenges trying to convince leadership about the need for more resources. Respondents suggested numerous ways that the CDC could communicate that OSH and WHP were core disciplines in public health. These included: 1) funding all states for at least basic OSH surveillance, so that OSH was not viewed as “elective”; 2) providing increased and consistent funding for WHP programming; 3) incorporating industry and occupational indicators into all federal public health surveys; 4) educating SHD leadership about the unique role of public health departments in protecting worker health (and how this differs from the role of Departments of Labor in each state); 5) encouraging SHDs to include worksite objectives in their State Healthy People plans, and 6) disseminating CDC-branded worker health-related tools and information that identifies worker health as a national public health priority.

Several study limitations are noteworthy. Our sampling method sought to identify individuals in the SHD who were “most knowledgeable” about the WHP or OSH activities being performed by the department, by drawing from relevant organizational contact lists. However, despite a high overall response rate and several checks with key professional groups/organizations, we cannot be certain our respondents were the most experienced or knowledgeable about OSH or WHP activities at their SHD. For OSH respondents, we compared our recruitment list to the Occupational Health Surveillance (OHS) Subcommittee of CSTE's point of contact list. From this we determined that there were only 3 individuals in our sample for which the CSTE OHS Subcommittee would have definitively listed a different person as “most knowledgeable” about OSH. We compared the activity data of these three respondents against the data from the remaining respondents who matched CSTE’s point of contact list, and found no consistent differences. Thus, given our overall response rate and checks on sample respondents, we believe our sample was representative.

Another limitation is the reliance on self-reported data for survey items on activities, capacity, funding, FTE, and activity estimates. This is a limitation of all surveys but we had expert review of the survey items upfront, pilot tested both the survey and interviews with SHDs before we launched the survey, and, for any data that were extreme values during cleaning, did follow-up with respondents to verify data. We also took the opportunity to check preliminary findings with the WHRN and a group of OSH professionals to improve the framing and interpretation of our results.

Finally, the activity scale only took into account the presence of activity, not the quality or reach of any particular activity. In fact, respondents from states with broad activity scores emphasized that “*there are a lot of opportunities…the reality is that we’re just barely scratching the surface on this work*” (WHP2). A multi-dimensional measure of activity (including type, number, reach and quality) would be desirable and could be the subject of future research. Our small sample size and relatively simple measure of activity may have contributed to our finding of few differences in interview results between respondents of different activity levels. Relatedly, because different survey items were used to assess activity for OSH vs. WHP respondents (e.g. follow-back investigations and standard/policy guidelines were only asked of OSH respondents), our findings cannot be used to establish whether SHDs are more or less active on OSH vs. WHP. Despite these limitations, our study revealed new and important information that has not been available previously regarding both WHP and OSH activities, current capacity and recommendations for improving capacity in SHDs in the US setting.

Strengths of this study were that all study instruments were developed by members of the Workplace Health Research Network [11], a research group with experts across six universities specializing with extensive experience in workplace health and safety research and practice. The survey had more than a 70% response rate from both OSH and WHP respondents and included respondents from SHDs with variation in the amount of activity and reported capacity levels. The mixed-methods approach allowed the team to clarify and expand upon survey results with qualitative interview data.

## Conclusions

In conclusion, these results document SHD activities and current capacity to conduct OSH and WHP activities. SHDs reported a wide range of activities despite facing limited funding, staffing, and organizational support for OSH and WHP. They were most active in surveillance for OSH and employer implementation support for WHP. More importantly, survey and in-depth interview results offer very specific, actionable recommendations for increasing SHDs’ capacity to promote health and safety among workers and workplaces. These include: consistently funding all SHDs to conduct OSH and WHP work; modifying current funding streams to focus on practice (rather than research) and to have greater flexibility in how funds are used; and building SHDs’ leaders’ awareness by releasing CDC-branded resources identifying worker health as a national priority. In addition, respondents encouraged the CDC and SHD leadership to find ways to integrate OSH and WHP staff and approaches into other public health initiatives within health departments, as this could improve the reach and quality of programs for all partners. Given the fact that most adults work and spend a large proportion of their day working, work as a social determinant of health needs wider recognition, action, and support at the state, local and national levels. We intend for these results and related recommendations to be a catalyst for more conversation and action on this important public health topic within US State and Territorial Health Departments, and among funders who support the activities that occur in these organizations.

## Additional files

Additional file 1: Survey Worksheet: National Survey of State and Territorial Health Departments’ Workplace Health and Safety Activities (OSH version). (PDF 218 kb)

Additional file 2: Survey Worksheet: National Survey of State and Territorial Health Departments’ Workplace Health and Safety Activities (WHP version). (PDF 242 kb)

Additional file 3: National Survey of State and Territorial Health Departments’ Workplace Health and Safety Activities: Follow-up Interview Questions (OSH version) (PDF 179 kb)

Additional file 4: Occupational Safety and Health (OSH) Surveillance Activities. The prevalence of specific OSH surveillance activities as reported by survey respondents. (PDF 100 kb)

Additional file 5: Implementation Supports – How Many Employers Are Reached?. Survey respondents’ report of how many employers receive OSH/WHP tools, trainings, technical assistance, and/or quality assurance/quality improvement from the health department. (PDF 98 kb)

## Abbreviations

ASTHO: Association of State and Territorial Health Officials
CDC: Centers for Disease Control and Prevention
CSTE: Council of State and Territorial Epidemiologists
EPA: US Environmental Protection Agency
NIOSH: National Institute for Occupational Safety and Health
OHI: Occupational Health Indicators
OSH: Occupational Safety and Health
SHDs: State and Territorial Health Departments
WHP: Workplace Health Promotion
WHRN: Workplace Health Research Network

## Footnotes

1 The CDC defines bona fide agents as: “organizations designated by the health department as eligible to submit a grant application in lieu of the health department. These organizations may simply apply for the grant and funnel money to the health department, or may undertake more of the grant activities, depending on the local situation.” (https://www.cdc.gov/stltpublichealth/grantsfunding/expediting.html#Q7)

2 In 2016, 26 of 50 states were funded through the NIOSH State Occupational Health and Safety Surveillance Program (https://www.cdc.gov/niosh/oep/statesurv.html). State Health Departments in these states collect data on 15–20 Occupational Health Indicators, develop partnerships with other OSH stakeholders in their state, develop prevention recommendations, and carry out targeted surveillance on emerging and priority issues.
