# Supplementary material for: Workplace health promotion and safety in state and territorial health departments in the United States: a national mixed-methods study of activity, capacity, and growth opportunities
Source: BMC Public Health. 2019 Mar 12;19:291. doi: 10.1186/s12889-019-6575-x (PMC6417036; doi:10.1186/s12889-019-6575-x)
Supplement: Supplementary file 1 — Survey Worksheet: National Survey of State and Territorial Health Departments’ Workplace Health and Safety Activities (OSH version). (PDF 218 kb) [file 12889_2019_6575_MOESM1_ESM.pdf]

Survey Worksheet: National Survey of State and Territorial Health Departments' Workplace Health and Safety Activities

This worksheet is intended to help you collect the data you will need to complete the national survey of State and Territorial Health Departments' Workplace Health and Safety Activities.

We would like to start with some questions about the occupational safety and health activities performed by, or on behalf of, your Health Department **in the last 12 months**.

In this study, occupational safety and health is defined as the use of programs, policies, and practices (including the hierarchy of hazard controls) to protect workers from work-related safety and health hazards and to promote workers' health.

For each of the activities listed below, please indicate if:

- The activity is performed **by your Health Department, independently**,
- The activity is performed by your Health Department **in partnership with another organization(s),**
- The activity is performed **by a "bona-fide agent,"** acting on behalf of your Health Department, or,
- To your knowledge, **your Health Department has not been involved in performing this activity during the last 12 months**.

You may select more than one response, if this activity is carried out differently in different projects/settings.

Occupational Safety and Health Surveillance Activities. The following activities are performed...(check all that apply)

	by your Health Department, independently.	in partnership with another organization(s)	by a "bona-fide agent," acting on behalf of your Health Department.	Your Health Department has not performed this activity in the last 12 months.
Compile, analyze, and interpret Occupational Health Indicators (OHIs) (see http://www.cste.org/?OHIndicators for a list of the OHIs)	❑	❑	❑	❑
Workers' Compensation surveillance, beyond the collection of Workers' Compensation-related OHIs	❑	❑	❑	❑

	by your Health Department, independently.	in partnership with another organization(s)	by a "bona-fide agent," acting on behalf of your Health Department.	Your Health Department has not performed this activity in the last 12 months.
Surveillance of occupational lead levels in adults and submission of data to the NIOSH Adult Blood Lead Epidemiology and Surveillance (ABLES) program	❑	❑	❑	❑
Monitoring data from the National Surveillance System for Pneumoconiosis Mortality (NSSPM)	❑	❑	❑	❑
Monitoring of indicators for the Healthy People 2020 occupational safety and health (OSH) objectives	❑	❑	❑	❑
Targeted surveillance for Fatality assessment, control, and evaluation (FACE)	❑	❑	❑	❑
Targeted surveillance of occupational respiratory disease	❑	❑	❑	❑
Targeted surveillance of pesticide illness and injury	❑	❑	❑	❑
Targeted surveillance of musculoskeletal disorders	❑	❑	❑	❑
Targeted surveillance of "target worker populations" (e.g., youth, older age workers, immigrant workers, temporary workers, and workers in high-risk industries/occupations).	❑	❑	❑	❑
Presentation of OSH surveillance data to relevant staff (occupational health professionals and health care providers) at workplaces within your state	❑	❑	❑	❑

If you indicate that you conduct surveillance of "target worker populations" in any capacity (independently, in partnership, or through a "bona-fide agent") you will see:

For which "target worker population(s)" do you conduct targeted surveillance? Please list:

____

If you indicate that you compile, analyze, and interpret OHIs in any capacity (independently, in partnership, or through a "bona-fide agent") you will see:

You indicated that your Health Department is involved in compiling, analyzing, and interpreting the Council of State and Territorial Epidemiologists (CSTE)-published Occupational Health Indicators (OHIs). What proportion of these 22 indicators are compiled, analyzed, and interpreted in your state? (See http://www.cste.org/?OHIndicators for a list of the OHIs)

- ❍ 14 or fewer indicators
- ❍ Between 15 and 21 indicators
- ❍ All 22 indicators

If you answer that you perform any of the occupational safety and health surveillance activities in ***partnership*** *with another organization, you will see the following three questions:*

With whom do you partner to perform these surveillance activities? (Check all that apply)

- ❑ Local Health Department
- ❑ Hospital or health system
- ❑ Voluntary health agency
- ❑ Health insurance company
- ❑ University
- ❑ Union
- ❑ Chamber of Commerce
- ❑ Broker
- ❑ Consultant
- ❑ Other State Agency, please specify ____________________
- ❑ Other, please specify ____________________

Please rate the overall effectiveness of this partnership(s) in meeting your Health Department's goals for performing occupational safety and health surveillance activities.

- ❍ Not effective at all
- ❍ Slightly effective
- ❍ Moderately effective
- ❍ Very effective
- ❍ Extremely effective

Please describe up to three factors that you feel influence the effectiveness of your partnerships in meeting your Health Department's goals for performing occupational safety and health surveillance activities:

All participants will see:
Does your state collect data about industry and occupation as part of the Behavioral Risk Factor Surveillance System (BRFSS) survey?

- ❍ Yes
- ❍ No

Please describe any additional Occupational Safety and Health surveillance activities that you conduct, beyond those listed in the previous questions. Include any surveillance activites carried out by your Health Department independently, in partnership with another organization(s), as well as those conducted by a "bona-fide agent." Do not include activities conducted by another state agency:

To your knowledge, are other state agencies in your state involved in occupational health and safety surveillance activities, independent of any work they do in partnership with you?

- ❍ Yes
- ❍ No
- ❍ Don't know

If yes, other state agencies are involved in OHS surveillance activities:
Please list these activities, to the best of your knowledge:

Occupational Safety & Health Follow-up Intervention & Prevention Activities. The following activities are performed...(check all that apply)

	by your Health Department, independently	in partnership with another organization(s)	by a "bona-fide agent," acting on behalf of your Health Department	Your Health Department has not performed this activity in the last 12 months.
Development and dissemination of educational materials to employers	❑	❑	❑	❑
Providing training and education programs to employers	❑	❑	❑	❑
Providing technical assistance to employers, upon request	❑	❑	❑	❑
Providing employers with assistance in monitoring the quality of their occupational safety and health programs, for example through providing organizational audits or other evaluation resources	❑	❑	❑	❑
Worksite follow-back investigations	❑	❑	❑	❑
Referrals of employers/worksites to OSHA or other agencies for follow-back investigations	❑	❑	❑	❑
Policy and standard development	❑	❑	❑	❑

For each of the follow-up intervention & prevention activities we ask about above, if you answer that you perform this activity in any capacity (independently, with a partner, through a "bona-fide agent"), you will be asked the following two questions:

Please estimate the number of employers who used these [follow-up intervention & prevention activities] during the past 12 months.

	0-10	11-25	26-50	51-100	101-250	251 or more	We do not track this information
Development and dissemination of educational materials to employers							
Providing training and education programs to employers							
Providing technical assistance to employers, upon request							
Providing employers with assistance in monitoring the quality of their occupational safety and health programs, for example through providing organizational audits or other evaluation resources							

Please provide a brief description of these [follow-up intervention & prevention activities] (up to 3 examples):

Development and dissemination of educational materials to employers	
Providing training and education programs to employers	
Providing technical assistance to employers, upon request	
Providing employers with assistance in monitoring the quality of their occupational safety and health programs, for example through providing organizational audits or other evaluation resources	
Policy and standard development	

For each of the follow-up intervention & prevention activities we ask about above, if you answer that you perform this activity in ***partnership*** *with another organization, you will see the following two questions:*

With whom do you partner to [perform this follow-up intervention & prevention activity]? (Check all that apply)

	Partners	
Development and dissemination of educational materials to employers	❑ Local Health Department<br>❑ Hospital or health system<br>❑ Voluntary health agency<br>❑ Health insurance company<br>❑ University<br>❑ Union	❑ Chamber of Commerce<br>❑ Broker<br>❑ Consultant<br>❑ Other State Agency, please specify ___________<br>❑ Other, please specify___________
Providing training and education programs to employers	❑ Local Health Department<br>❑ Hospital or health system<br>❑ Voluntary health agency<br>❑ Health insurance company<br>❑ University<br>❑ Union	❑ Chamber of Commerce<br>❑ Broker<br>❑ Consultant<br>❑ Other State Agency, please specify ___________<br>❑ Other, please specify___________
Providing technical assistance to employers, upon request	❑ Local Health Department<br>❑ Hospital or health system<br>❑ Voluntary health agency<br>❑ Health insurance company<br>❑ University<br>❑ Union	❑ Chamber of Commerce<br>❑ Broker<br>❑ Consultant<br>❑ Other State Agency, please specify ___________<br>❑ Other, please specify___________
Providing employers with assistance in monitoring the quality of their occupational safety and health programs, for example through providing organizational audits or other evaluation resources	❑ Local Health Department<br>❑ Hospital or health system<br>❑ Voluntary health agency<br>❑ Health insurance company<br>❑ University<br>❑ Union	❑ Chamber of Commerce<br>❑ Broker<br>❑ Consultant<br>❑ Other State Agency, please specify ___________<br>❑ Other, please specify___________
Policy and standard development	❑ Local Health Department<br>❑ Hospital or health system<br>❑ Voluntary health agency<br>❑ Health insurance company<br>❑ University<br>❑ Union	❑ Chamber of Commerce<br>❑ Broker<br>❑ Consultant<br>❑ Other State Agency, please specify ___________<br>❑ Other, please specify___________

Please rate the overall effectiveness of this partnership(s) in meeting your Health Department's goals for [performing this follow-up intervention & prevention activity]

Development and dissemination of educational materials to employers	❍ Not effective at all<br>❍ Slightly effective<br>❍ Moderately effective<br>❍ Very effective<br>❍ Extremely effective
Providing training and education programs to employers	❍ Not effective at all<br>❍ Slightly effective<br>❍ Moderately effective<br>❍ Very effective<br>❍ Extremely effective
Providing technical assistance to employers, upon request	❍ Not effective at all<br>❍ Slightly effective<br>❍ Moderately effective<br>❍ Very effective<br>❍ Extremely effective
Providing employers with assistance in monitoring the quality of their occupational safety and health programs, for example through providing organizational audits or other evaluation resources	❍ Not effective at all<br>❍ Slightly effective<br>❍ Moderately effective<br>❍ Very effective<br>❍ Extremely effective
Policy and standard development	❍ Not effective at all<br>❍ Slightly effective<br>❍ Moderately effective<br>❍ Very effective<br>❍ Extremely effective

You will also be asked one general question about all of your partnerships for follow-up intervention and prevention activities:

Please describe up to three factors that you feel influence the effectiveness of your partnerships in meeting your Health Department's goals for performing occupational safety & health follow-up intervention & prevention activities:

Finally, if you answer that you provide educational materials, trainings, and/or technical assistance in any capacity (independently, with a partner, or through a "bona-fide" agent), you will be asked the following question:

In the **past 12 months**, have you performed any Quality Assurance/Quality Improvement activities (whether independently, in partnership with another organization, or through a "bona-fide agent") to monitor and improve the educational materials, trainings, and/or technical assistance that **your Health Department provided to employers?**

Include things such as:

- tracking occupational safety & health-relevant outcomes among employers who use the educational materials, trainings and technical assistance, or,
- tracking process indicators, such as employer satisfaction with the educational materials, trainings, and technical assistance.

❍ Yes
❍ No

All participants will see this final question about follow-up intervention & prevention activities:
To your knowledge, are other state agencies in your state involved in occupational health and safety follow-up intervention & prevention activities, independent of any work they do in partnership with you?
❍ Yes
❍ No
❍ Don't know

If yes:
Please list these activities, to the best of your knowledge:

Direct Service Activities: delivering occupational safety & health services directly to workers. The following activity is performed...(check all that apply)

	by your Health Department, independently	in partnership with another organization(s)	by a "bona-fide agent," acting on behalf of your Health Department	Your Health Department has not performed this activity in the last 12 months
Providing educational materials, training, and/or technical assistance **to workers directly**	❑	❑	❑	❑

If you answer that you peform direct service activities in any capacity (independently, with a partner, through a "bona-fide agent"), you will be asked the following two questions:

Please estimate the number of workers who used these direct services during the past 12 months.
❍ 0-100
❍ 101-250
❍ 251-500
❍ 501-1000
❍ 1001-2500
❍ 2501 or more
❍ We do not track this information

Please provide a brief description of the educational materials, training, and/or technical assistance that is provided to workers directly (up to 3 examples):

If you answer that you ***partner*** *with another organization to perform the direct service activities, you will see three follow-up questions:*

With whom do you partner to provide these direct services to workers? (Check all that apply)

- ❑ Local Health Department
- ❑ Hospital or health system
- ❑ Voluntary health agency
- ❑ Health insurance company
- ❑ University
- ❑ Union
- ❑ Chamber of Commerce
- ❑ Broker
- ❑ Consultant
- ❑ Other State Agency, please specify ___________________
- ❑ Other, please specify ___________________

Please rate the overall effectiveness of this/these partnership(s) in meeting your Health Department's goals for providing educational materials, training, and/or technical assistance to workers directly.

- ❍ Not effective at all
- ❍ Slightly effective
- ❍ Moderately effective
- ❍ Very effective
- ❍ Extremely effective

Please describe up to three factors that you feel influence the effectiveness of your partnerships in meeting your Health Department's goals for providing educational materials, training, and/or technical assistance to workers directly:

All participants will see this final question about direct service activities:

To your knowledge, are other state agencies in your state involved in direct occupational health and safety service activities, independent of any work they do in partnership with you?

- ❍ Yes
- ❍ No
- ❍ Don't know

If yes:

Please list these activities, to the best of your knowledge:

Small Employers

Please describe any occupational safety and health surveillance, follow-up intervention & prevention, and direct service activities that specifically target small employers (those with less than 250 employees):

We would now like to ask you a series of questions about your Health Department's resources and capacity to perform Occupational Safety and Health activities.

Human Resources

Please indicate the total number of FTEs (to the nearest 0.1 FTE) performing occupational safety and health activities in your Health Department during the **past 12 months**. Include all those employed by the state, all those working at the state-level who are either federal assignees or contract employees, and state employees assigned to work in a regional office.

Total number of FTEs: ____

Approximately what percentage of these FTEs are funded by state funds?

% of FTEs funded by state funds: ____

Financial Resources

Please estimate your Health Department's total funding for occupational safety and health activities during the **past 12 months**, including federal, state, and other funds.

$ ____

Approximately what percentage of these funds are from state funds?

% from state funds: ____

Competency

Please indicate your Health Department's level of competency (knowledge & skills) to perform the following occupational safety and health activities:

	Minimal or none	Basic	Intermed-iate	Advanced	Expert
Occupational safety and health surveillance activities	❍	❍	❍	❍	❍
Follow-up intervention and prevention activities	❍	❍	❍	❍	❍
Direct service activities: delivering occupational safety & health services directly to employees	❍	❍	❍	❍	❍

What specific types of support or training would your Health Department need to increase your competency (knowledge & skills) to perform occupational safety and health activities?:

Occupational Safety and Health Resources

Please indicate your Health Department's awareness and utilization of the following occupational safety and health resources. For each of the following, is your Health Department:

- **Not aware** of the resource,
- **Aware** of the resource, **but has not utilized it**,
- **Aware** of the resource, **and has utilized it.**

	Not aware	Aware, but has not utilized	Aware, and has utilized
Resources from the National Institute of Occupational Safety and Health (NIOSH), such as meetings, trainings, and educational materials	❍	❍	❍
Resources from the Council of State and Territorial Epidemiologists (CSTE), such as meetings, trainings, and educational materials	❍	❍	❍
Resources from the Occupational Safety and Health Administration (OSHA), such as meetings, trainings, and educational materials	❍	❍	❍

Please briefly describe any other helpful occupational safety and health resources (including meetings, trainings, and educational materials) that your Health Department has used in the past 12 months:

What resources (drawing from both the list provided two questions above, as well as any additional resources which you described) have been most helpful to your Health Department in performing occupational safety and health work? Please be as specific as possible, name up to three.

Resource 1: ______________________________

Resource 2: ______________________________

Resource 3: ______________________________

Overall Commitment and Capacity

Please rate your Health Department's commitment to occupational safety and health.

- ❍ Not at all committed
- ❍ Slightly committed
- ❍ Moderately committed
- ❍ Very committed
- ❍ Extremely committed

What level of priority does your Health Department assign to occupational safety and health efforts, in comparison to other efforts your Health Department is involved in?

- ❍ This is not a priority for my Health Department
- ❍ This is a low level priority for my Health Department
- ❍ This is a moderate level priority for my Health Department
- ❍ This is a high level priority for my Health Department
- ❍ This is a very high level priority for my Health Department

Please rate your Health Department's overall capacity to support occupational safety and health among employers in the state.

- ❍ No capacity
- ❍ Minimal capacity
- ❍ Some capacity
- ❍ Moderate capacity
- ❍ Substantial capacity

What specific types of assistance would your Health Department need to increase your capacity to support occupational safety and health? Consider aspects such as: human resources, financial resources, data resources, partnerships, and organizational culture, structure, and leadership.

The following questions are about collaboration between Occupational Safety & Health staff and Workplace Health Promotion staff in your Health Department.

Please rate your level of knowledge about workplace health promotion activities being performed by your Health Department.
- ❍ Know nothing
- ❍ Know some
- ❍ Know a moderate amount
- ❍ Know a substantial amount
- ❍ Extremely knowledgable

Do you ever collaborate with workplace health promotion staff in your Health Department on workplace safety and/or health promotion activities?
- ❍ Yes
- ❍ No

*If you answer **yes, you do collaborate**, you will see the following four questions:*
Please rate the level of collaboration between occupational safety and health and workplace health promotion staff in your Health Department on workplace safety and health promotion activities.
- ❍ Very low, we engage in limited collaborative projects and/or provide limited support to one another.
- ❍ Low
- ❍ Moderate, we work on some collaborative projects and/or provide some support to one another.
- ❍ High
- ❍ Very high, we work closely on collaborative projects and/or provide a great deal of support to one another.

Please describe one example of how you collaborate:

Please rate your level of agreement with the following statements, from strongly disagree to strongly agree:
Collaboration between occupational safety and health and workplace health promotion staff helps my Health Department achieve our aims for workplace safety and health promotion.
❍ Strongly disagree
❍ Somewhat disagree
❍ Neither agree nor disagree
❍ Somewhat agree
❍ Strongly agree

Collaboration between occupational safety and health and workplace health promotion staff in my Health Department is easy.
❍ Strongly disagree
❍ Somewhat disagree
❍ Neither agree nor disagree
❍ Somewhat agree
❍ Strongly agree

*If you answer **no, you do not collaborate**, you will see the following three questions:*
Please rate your level of agreement with the following statements, from strongly disagree to strongly agree:
Collaboration between occupational safety and health and workplace health promotion staff would help my Health Department achieve our aims for workplace safety and health promotion.
❍ Strongly disagree
❍ Somewhat disagree
❍ Neither agree nor disagree
❍ Somewhat agree
❍ Strongly agree

Collaboration between occupational safety and health and workplace health promotion staff in my Health Department would be easy.
❍ Strongly disagree
❍ Somewhat disagree
❍ Neither agree nor disagree
❍ Somewhat agree
❍ Strongly agree

Please describe the top three challenges to effective collaboration between occupational safety and health and workplace health promotion staff in your Health Department:

All participants will see the following collaboration question:
Do you collaborate with staff from any of the following public health programs in your Health Department?

	Yes	No
Infectious disease program	❍	❍
Injury and violence prevention and control program	❍	❍
Cancer prevention and control program	❍	❍
Asthma program	❍	❍
Women's and children's health program	❍	❍
Other chronic disease prevention and control program(s), please describe: ______________________________	❍	❍
Other types of programs in your Health Department, please describe: ______________________________	❍	❍

The next few questions are about your Health Department's familiarity with "Total Worker Health". Total Worker Health is a strategy supported by the Centers for Disease Control and Prevention's National Institute for Occupational Safety and Health (NIOSH) that integrates occupational safety and health protection with health promotion to prevent worker injury and illness and to advance worker health and well-being.

Please rate your level of familiarity with NIOSH's Total Worker Health initiative.

- ❍ Not familiar at all
- ❍ Slightly familiar
- ❍ Moderately familiar
- ❍ Very familiar
- ❍ Extremely familiar

Please rate your level of agreement with the following statement, from strongly disagree to strongly agree.
Applying an integrated approach to workplace safety and workplace health promotion is a priority for employers in my state.

- ❍ Strongly disagree
- ❍ Somewhat disagree
- ❍ Neither agree nor disagree
- ❍ Somewhat agree
- ❍ Strongly agree

Please indicate your Health Department's awareness and utilization of the following Total Worker Health-related resources:

	Not aware	Aware, but has not utilized	Aware, and has utilized
The NIOSH Total Worker Health webinar series	❍	❍	❍
The NIOSH Total Worker Health in Action! eNewsletter	❍	❍	❍
The "SafeWell Practice Guidelines: an Integrated Approach to Worker Health v2.0" from the Harvard School of Public Health's Center for Work, Health, & Well-Being	❍	❍	❍
The "Healthy Workplace Participatory Program" online toolkit from the Center for the Promotion of Health in the New England Workplace	❍	❍	❍
"The Whole Worker: Guidelines for Integrating Occupational Health and Safety with Workplace Wellness Programs" from the State of California's Commission on Health and Safety and Workers' Compensation	❍	❍	❍
The "Indicators of Integration" assessment tool from the Harvard School of Public Health's Center for Work, Health, & Well-Being	❍	❍	❍
The "Corporate Health Achievement Award self-assessment" from the American College of Occupational and Environmental Medicine	❍	❍	❍
The Journal of Occupational and Environmental Medicine's Total Worker Health Supplement (Vol 55, Supplement 12S, Dec 2013)	❍	❍	❍
The "CPH News & Views" bi-monthly emerging topics briefs from the Center for the Promotion of Health in the New England Workplace	❍	❍	❍
The "Total Worker Health Essentials" video series from the University of Iowa's Healthier Workforce Center for Excellence	❍	❍	❍

What resources (drawing from both the list provided above, as well as any additional resources which you are aware of) have been most helpful to your Health Department in performing integrated workplace safety and health promotion work? Please be as specific as possible, name up to three.

Resource 1: ______________________________

Resource 2: ______________________________

Resource 3: ______________________________

We would now like to ask some questions about how occupational safety and health is seen by different stakeholders in your state. Please rate your level of agreement with the following statements, from strongly disagree to strongly agree.

Improving worker health and safety is a priority for employers in my state.

- ❍ Strongly disagree
- ❍ Somewhat disagree
- ❍ Neither agree nor disagree
- ❍ Somewhat agree
- ❍ Strongly agree

Improving worker health and safety is a priority for labor unions in my state.

- ❍ Strongly disagree
- ❍ Somewhat disagree
- ❍ Neither agree nor disagree
- ❍ Somewhat agree
- ❍ Strongly agree

Improving worker health and safety is a priority for legislators in my state.

- ❍ Strongly disagree
- ❍ Somewhat disagree
- ❍ Neither agree nor disagree
- ❍ Somewhat agree
- ❍ Strongly agree

Improving worker health and safety is a priority for the governor in my state.

- ❍ Strongly disagree
- ❍ Somewhat disagree
- ❍ Neither agree nor disagree
- ❍ Somewhat agree
- ❍ Strongly agree

Interviews

Over the summer, the research team at the University of North Carolina Gillings School of Global Public Health will be conducting interviews with selected State Health Departments to gain a deeper understanding of workplace safety and health promotion activities and capacity. Do we have your permission to contact you about participating in a follow-up interview?

- ❍ Yes
- ❍ No
