# Supplementary material for: Workplace health promotion and safety in state and territorial health departments in the United States: a national mixed-methods study of activity, capacity, and growth opportunities
Source: BMC Public Health. 2019 Mar 12;19:291. doi: 10.1186/s12889-019-6575-x (PMC6417036; doi:10.1186/s12889-019-6575-x)
Supplement: Supplementary file 2 — Survey Worksheet: National Survey of State and Territorial Health Departments’ Workplace Health and Safety Activities (WHP version). (PDF 242 kb) [file 12889_2019_6575_MOESM2_ESM.pdf]

# Survey Worksheet: National Survey of State and Territorial Health Departments' Workplace Health and Safety Activities

This worksheet is intended to help you collect the data you will need to complete the national survey of State and Territorial Health Departments' Workplace Health and Safety Activities.

We would like to start with some questions about the workplace health promotion activities performed by, or on behalf of, your Health Department **in the last 12 months**.

In this study, workplace health promotion is defined as the use of workplace programs, policies, and environmental supports to support healthy behaviors and improve employee health. We will be asking about your activities with employers and workers across your state. Except where specifically indicated, we will not be asking about programs offered to employees of your Health Department.

For each of the activities listed in the next few pages, please indicate if:

- The activity is performed **by your Health Department, independently**,
- The activity is performed by your Health Department **in partnership with another organization(s)**,
- The activity is performed **by another organization(s) on behalf of your Health Department**, and the Health Department contracts and pays this organization for this work, or,
- To your knowledge, **your Health Department has not been involved in performing this activity in the last 12 months**.

You may select more than one response, if this activity is carried out differently in different projects or settings.

**Workplace Health Promotion Surveillance Activities.** The following activities are performed...(check all that apply)

| | by your Health Department, independently. | in partnership with another organization(s). | by another organization(s) on behalf of your Health Department. | Your Health Department has not performed this activity in the last 12 months. |
|---|---|---|---|---|
| Monitoring of Healthy People 2020 worksite-related objectives? | ❑ | ❑ | ❑ | ❑ |
| Monitoring of other workplace health promotion activities in the state, beyond the Healthy People 2020 objectives (please describe)______________________ | ❑ | ❑ | ❑ | ❑ |

*For either of the two surveillance activities above, if you indicated that you work in* ***partnership*** *with another organization, you will be asked the following two questions:*

With whom do you partner to monitor these objectives? (Check all that apply)

| | Partners | |
|---|---|---|
| Monitoring of Healthy People 2020 worksite-related objectives | ❑ Local Health Department<br>❑ Hospital or health system<br>❑ Voluntary health agency<br>❑ Health insurance company<br>❑ University<br>❑ Union | ❑ Chamber of Commerce<br>❑ Broker<br>❑ Consultant<br>❑ Other State Agency, please specify __________________<br>❑ Other, please specify __________________ |
| Monitoring of other workplace health promotion activities in the state, beyond the Healthy People 2020 objectives | ❑ Local Health Department<br>❑ Hospital or health system<br>❑ Voluntary health agency<br>❑ Health insurance company<br>❑ University<br>❑ Union | ❑ Chamber of Commerce<br>❑ Broker<br>❑ Consultant<br>❑ Other State Agency, please specify __________________<br>❑ Other, please specify __________________ |

Please rate the effectiveness of this partnership(s) in meeting your Health Department's goals for:

| | |
|---|---|
| Monitoring of Healthy People 2020 worksite-related objectives. | ❍ Not effective at all<br>❍ Slightly effective<br>❍ Moderately effective<br>❍ Very effective<br>❍ Extremely effective |
| Monitoring of other workplace health promotion activities in the state, beyond the Healthy People 2020 objectives. | ❍ Not effective at all<br>❍ Slightly effective<br>❍ Moderately effective<br>❍ Very effective<br>❍ Extremely effective |

*You will also be asked one general question about all of your partnerships:*

Please describe up to three factors that you feel influence the effectiveness of your partnerships in meeting your Health Department's goals for workplace health promotion surveillance activities.

*For each of the two surveillance activities we ask about above, if you answer that you* ***contract*** *with another organization, you will see two follow-up questions:*

With whom do you contract to monitor these objectives? (Check all that apply)

| | Partners | |
|---|---|---|
| Monitoring of Healthy People 2020 worksite-related objectives | ❑ Local Health Department<br>❑ Hospital or health system<br>❑ Voluntary health agency<br>❑ Health insurance company<br>❑ University<br>❑ Union | ❑ Chamber of Commerce<br>❑ Broker<br>❑ Consultant<br>❑ Other State Agency, please specify ____________<br>❑ Other, please specify ____________ |
| Monitoring of other workplace health promotion activities in the state, beyond the Healthy People 2020 objectives | ❑ Local Health Department<br>❑ Hospital or health system<br>❑ Voluntary health agency<br>❑ Health insurance company<br>❑ University<br>❑ Union | ❑ Chamber of Commerce<br>❑ Broker<br>❑ Consultant<br>❑ Other State Agency, please specify ____________<br>❑ Other, please specify ____________ |

Please rate the overall performance of this organization(s) in meeting your Health Department's goals for:

| | |
|---|---|
| Monitoring of Healthy People 2020 worksite-related objectives. | ❍ Not effective at all<br>❍ Slightly effective<br>❍ Moderately effective<br>❍ Very effective<br>❍ Extremely effective |
| Monitoring of other workplace health promotion activities in the state, beyond the Healthy People 2020 objectives. | ❍ Not effective at all<br>❍ Slightly effective<br>❍ Moderately effective<br>❍ Very effective<br>❍ Extremely effective |

*You will also be asked one general question about all of your contracts:*

Please describe up to three factors that you feel influence the effectiveness of your contracts in meeting your Health Department's goals for workplace health promotion surveillance activities.

**Implementation Support Activities: providing support to employers with the design and implementation of workplace health promotion initiatives**. The following activities are performed...(check all that apply)

| | by your Health Department, independently. | in partnership with another organization(s). | by another organization(s) on behalf of your Health Department. | Your Health Department has not performed this activity in the last 12 months. |
|---|---|---|---|---|
| Providing educational materials, resources and/or toolkits to employers | ❑ | ❑ | ❑ | ❑ |
| Providing training to employers, such as training sessions and/or webinars designed to introduce a group of employers to a workplace health promotion topic | ❑ | ❑ | ❑ | ❑ |
| Providing technical assistance to employers, such as individualized, on-demand consultations and/or phone-based support | ❑ | ❑ | ❑ | ❑ |
| Providing employers with assistance in monitoring the quality of their workplace health promotion programs, for example through providing organizational audits, health assessments, and/or other evaluation resources | ❑ | ❑ | ❑ | ❑ |

*For each of the implementation support activities we ask about above, if you answer that you perform this activity in any capacity (independently, with a partner, through a contracting organization), you will be asked the following two questions*:

Please estimate the number of employers who used these [implementation support activities] during the past 12 months.

| | 0-10 | 11-25 | 26-50 | 51-100 | 101-250 | 251 or more | We do not track this information |
|---|---|---|---|---|---|---|---|
| Providing educational materials, resources and/or toolkits to employers | | | | | | | |
| Providing training to employers, such as training sessions and/or webinars designed to introduce a group of employers to a workplace health promotion topic | | | | | | | |
| Providing technical assistance to employers, such as individualized, on-demand consultations and/or phone-based support | | | | | | | |
| Providing employers with assistance in monitoring the quality of their workplace health promotion programs, for example through providing organizational audits, health assessments, and/or other evaluation resources | | | | | | | |

Please provide a brief description of these [implementation support activities] (up to 3 examples):

| | |
|---|---|
| Providing educational materials, resources and/or toolkits to employers | |
| Providing training to employers, such as training sessions and/or webinars designed to introduce a group of employers to a workplace health promotion topic | |
| Providing technical assistance to employers, such as individualized, on-demand consultations and/or phone-based support | |
| Providing employers with assistance in monitoring the quality of their workplace health promotion programs, for example through providing organizational audits, health assessments, and/or other evaluation resources | |

*For each of the implementation support activities we ask about above, if you answer that you perform this activity in* ***partnership*** *with another organization, you will see the following two questions:*

With whom do you partner to [carry out this implementation support activity]?

| | Partners | |
|---|---|---|
| Providing educational materials, resources and/or toolkits to employers | ❑ Local Health Department<br>❑ Hospital or health system<br>❑ Voluntary health agency<br>❑ Health insurance company<br>❑ University<br>❑ Union | ❑ Chamber of Commerce<br>❑ Broker<br>❑ Consultant<br>❑ Other State Agency, please specify ___________<br>❑ Other, please specify ___________ |
| Providing training to employers, such as training sessions and/or webinars designed to introduce a group of employers to a workplace health promotion topic | ❑ Local Health Department<br>❑ Hospital or health system<br>❑ Voluntary health agency<br>❑ Health insurance company<br>❑ University<br>❑ Union | ❑ Chamber of Commerce<br>❑ Broker<br>❑ Consultant<br>❑ Other State Agency, please specify ___________<br>❑ Other, please specify ___________ |
| Providing technical assistance to employers, such as individualized, on-demand consultations and/or phone-based support | ❑ Local Health Department<br>❑ Hospital or health system<br>❑ Voluntary health agency<br>❑ Health insurance company<br>❑ University<br>❑ Union | ❑ Chamber of Commerce<br>❑ Broker<br>❑ Consultant<br>❑ Other State Agency, please specify ___________<br>❑ Other, please specify ___________ |
| Providing employers with assistance in monitoring the quality of their workplace health promotion programs, for example through providing organizational audits, health assessments, and/or other evaluation resources | ❑ Local Health Department<br>❑ Hospital or health system<br>❑ Voluntary health agency<br>❑ Health insurance company<br>❑ University<br>❑ Union | ❑ Chamber of Commerce<br>❑ Broker<br>❑ Consultant<br>❑ Other State Agency, please specify ___________<br>❑ Other, please specify ___________ |

Please rate the effectiveness of this partnership(s) in meeting your Health Department's goals for [carrying out this implementation support activity].

| | |
|---|---|
| Providing educational materials, resources and/or toolkits to employers | ❍ Not effective at all<br>❍ Slightly effective<br>❍ Moderately effective<br>❍ Very effective<br>❍ Extremely effective |
| Providing training to employers, such as training sessions and/or webinars designed to introduce a group of employers to a workplace health promotion topic | ❍ Not effective at all<br>❍ Slightly effective<br>❍ Moderately effective<br>❍ Very effective<br>❍ Extremely effective |
| Providing technical assistance to employers, such as individualized, on-demand consultations and/or phone-based support | ❍ Not effective at all<br>❍ Slightly effective<br>❍ Moderately effective<br>❍ Very effective<br>❍ Extremely effective |
| Providing employers with assistance in monitoring the quality of their workplace health promotion programs, for example through providing organizational audits, health assessments, and/or other evaluation resources | ❍ Not effective at all<br>❍ Slightly effective<br>❍ Moderately effective<br>❍ Very effective<br>❍ Extremely effective |

*You will also be asked one general question about all of your partnerships:*

Please describe up to three factors that you feel influence the effectiveness of your partnerships in meeting your Health Department's goals for workplace health promotion implementation support activities.

*For each of the four implementation support activities we ask about above, if you answer that you* ***contract*** *with another organization who performs this activity on behalf of your Health Department, you will see the following two questions:*

With whom do you contract to [carry out this implementation support activity]? (Check all that apply)

| | Partners | |
|---|---|---|
| Providing educational materials, resources and/or toolkits to employers | ❑ Local Health Department<br>❑ Hospital or health system<br>❑ Voluntary health agency<br>❑ Health insurance company<br>❑ University<br>❑ Union | ❑ Chamber of Commerce<br>❑ Broker<br>❑ Consultant<br>❑ Other State Agency, please specify ________________<br>❑ Other, please specify ________________ |
| Providing training to employers, such as training sessions and/or webinars designed to introduce a group of employers to a workplace health promotion topic | ❑ Local Health Department<br>❑ Hospital or health system<br>❑ Voluntary health agency<br>❑ Health insurance company<br>❑ University<br>❑ Union | ❑ Chamber of Commerce<br>❑ Broker<br>❑ Consultant<br>❑ Other State Agency, please specify ________________<br>❑ Other, please specify ________________ |
| Providing technical assistance to employers, such as individualized, on-demand consultations and/or phone-based support | ❑ Local Health Department<br>❑ Hospital or health system<br>❑ Voluntary health agency<br>❑ Health insurance company<br>❑ University<br>❑ Union | ❑ Chamber of Commerce<br>❑ Broker<br>❑ Consultant<br>❑ Other State Agency, please specify ________________<br>❑ Other, please specify ________________ |
| Providing employers with assistance in monitoring the quality of their workplace health promotion programs, for example through providing organizational audits, health assessments, and/or other evaluation resources | ❑ Local Health Department<br>❑ Hospital or health system<br>❑ Voluntary health agency<br>❑ Health insurance company<br>❑ University<br>❑ Union | ❑ Chamber of Commerce<br>❑ Broker<br>❑ Consultant<br>❑ Other State Agency, please specify ________________<br>❑ Other, please specify ________________ |

Please rate the overall performance of this organization(s) in meeting your Health Department's goals for [carrying out this implementation support activity].

| | |
|---|---|
| Providing educational materials, resources and/or toolkits to employers | ❍ Not effective at all<br>❍ Slightly effective<br>❍ Moderately effective<br>❍ Very effective<br>❍ Extremely effective |
| Providing training to employers, such as training sessions and/or webinars designed to introduce a group of employers to a workplace health promotion topic | ❍ Not effective at all<br>❍ Slightly effective<br>❍ Moderately effective<br>❍ Very effective<br>❍ Extremely effective |
| Providing technical assistance to employers, such as individualized, on-demand consultations and/or phone-based support | ❍ Not effective at all<br>❍ Slightly effective<br>❍ Moderately effective<br>❍ Very effective<br>❍ Extremely effective |
| Providing employers with assistance in monitoring the quality of their workplace health promotion programs, for example through providing organizational audits, health assessments, and/or other evaluation resources | ❍ Not effective at all<br>❍ Slightly effective<br>❍ Moderately effective<br>❍ Very effective<br>❍ Extremely effective |

*You will also be asked one general question about all of your contracts:*

Please describe up to three factors that you feel influence the effectiveness of your contracts in meeting your Health Department's goals for workplace health promotion implementation support activities.

*Finally, if you answer that you provide educational materials, trainings, and/or technical assistance in any capacity (independently, with a partner, through a contracting organization), you will be asked the following question:*

In the **past 12 months**, have you, your partner organizations, or your contracting organizations performed any Quality Assurance/Quality Improvement activities to monitor and improve the educational materials, trainings, and/or technical assistance that **you, your partners, or your contracting organizations provide to employers?**

Include things such as:

- tracking workplace health-relevant outcomes among employers who use the educational materials, trainings and technical assistance,
- tracking process indicators, such as employer satisfaction with the educational materials, trainings, and technical assistance.

❍ Yes
❍ No

**Direct Service Activities: delivering workplace health promotion services directly to workers.** The following activity is performed...(check all that apply)

| | by your Health Department, independently. | in partnership with another organization(s). | by another organization on behalf of your Health Department. | Your Health Department has not performed this activity in the last 12 months. |
|---|---|---|---|---|
| Delivering health promotion programs directly to workers across your state, such as flu shots or blood pressure screening programs | ❑ | ❑ | ❑ | ❑ |

*If you answer that you peform direct service activities in any capacity (independently, with a partner, through a contracting organization), you will be asked the following two questions:*

| | 0-100 | 101-250 | 251-500 | 501-1000 | 1001-2500 | 2501 or more | We donot track this information |
|---|---|---|---|---|---|---|---|
| Delivering health promotion programs directly to workers across your state, such as flu shots or blood pressure screening programs | | | | | | | |

Please estimate the number of workers who received these programs during the past 12 months.

Please provide a brief description of the health promotion programs that are provided directly to

| | |
|---|---|
| Delivering health promotion programs directly to workers across your state, such as flu shots or blood pressure screening programs | |

workers across your state (up to 3 examples):

*If you answer that you* ***partner*** *with another organization to perform the direct service activities, you will see three follow-up questions:*

With whom do you partner to deliver health promotion programs directly to workers across your state? (Check all that apply)

- ❑ Local Health Department
- ❑ Hospital or health system
- ❑ Voluntary health agency
- ❑ Health insurance company
- ❑ University
- ❑ Union
- ❑ Chamber of Commerce
- ❑ Broker
- ❑ Consultant
- ❑ Other State Agency, please specify ___________________
- ❑ Other, please specify ___________________

Please rate the effectiveness of this partnership(s) in meeting your Health Department's goals for delivering health promotion programs directly to workers across your state.

- ❍ Not effective at all
- ❍ Slightly effective
- ❍ Moderately effective
- ❍ Very effective
- ❍ Extremely effective

*You will also be asked one general question about all of your partnerships:*

Please describe up to three factors that you feel influence the effectiveness of your partnerships in meeting your Health Department's goals for delivering health promotion programs directly to workers across your state.

*If you answer that you **contract** with another organization to perform the direct service activities, you will see two follow-up questions:*

With whom do you contract to deliver health programs directly to workers across your state? (Check all that apply)

- ❑ Local Health Department
- ❑ Hospital or health system
- ❑ Voluntary health agency
- ❑ Health insurance company
- ❑ University
- ❑ Union
- ❑ Chamber of Commerce
- ❑ Broker
- ❑ Consultant
- ❑ Other State Agency, please specify ___________________
- ❑ Other, please specify ___________________

Please rate the overall performance of this organization(s) in meeting your Health Department's goals for delivering health promotion programs directly to workers across your state.

- ❍ Not effective at all
- ❍ Slightly effective
- ❍ Moderately effective
- ❍ Very effective
- ❍ Extremely effective

*You will also be asked one general question about all of your contracts:*
Please describe up to three factors that you feel influence the effectiveness of your contracts in meeting your Health Department's goals for delivering health promotion programs directly to workers across your state.

**Small employers**
Please describe any workplace health promotion surveillance, implementation support, or direct service activities that specifically target small employers (those with less than 250 employees).

**We would now like to ask you a series of questions about your Health Department's resources and capacity to perform Workplace Health Promotion activities.**

**Human Resources**

Please indicate the total number of FTEs (to the nearest 0.1 FTE) performing workplace health promotion activities in your Health Department during the **past 12 months**. Include all those employed by the state, all those working at the state-level who are either federal assignees or contract employees, and state employees assigned to work in a regional office.

Total number of FTEs: ______________________________

Approximately what percentage of these FTEs are funded by state funds?

% of FTEs funded by state funds: ______________________________

**Financial Resources**

Please estimate your Health Department's total funding for workplace health promotion activities during the **past 12 months**, including federal, state, and other funds.

$ ______________________________

Approximately what percentage of these funds are from state funds?

% from state funds: ______________________________

Is any 1422 funding being allocated to workplace health promotion activities in your Health Department?

❍ Yes
❍ No
❍ Don't know

**Competency**

Please indicate your Health Department's level of competency (knowledge & skills) to perform the following workplace health promotion activities:

| | Minimal or none | Basic | Intermed-iate | Advanced | Expert |
|---|---|---|---|---|---|
| Workplace health promotion surveillance activities | ❍ | ❍ | ❍ | ❍ | ❍ |
| Implementation support activities: providing support to employers with design and implementation of workplace health promotion initiatives. | ❍ | ❍ | ❍ | ❍ | ❍ |
| Direct service activities: delivering workplace health promotion services directly to employees across your state. | ❍ | ❍ | ❍ | ❍ | ❍ |

What specific types of support or training would your Health Department need to increase your competency (knowledge & skills) to perform workplace health promotion activities?:

**Workplace Health Promotion Resources**

Please indicate your Health Department's awareness and utilization of workplace health promotion resources. For each of the following, is your Health Department:

- **Not aware** of the resource,
- **Aware** of the resource, **but has not utilized it,**
- **Aware** of the resource, **and has utilized it.**

| | Not aware | Aware, but has not utilized | Aware, and has utilized |
|---|---|---|---|
| CDC Worksite Health Scorecard | ❍ | ❍ | ❍ |
| CDC Work@Health® training program | ❍ | ❍ | ❍ |
| CDC Workplace Health Promotion Website, including resources on how to design, implement, and evaluate effective workplace health programs | ❍ | ❍ | ❍ |
| National Diabetes Education Program (NDEP) resources, such as the Diabetes at Work website | ❍ | ❍ | ❍ |
| American Cancer Society (ACS) resources, such as the ACS Workplace Health Assessment | ❍ | ❍ | ❍ |
| American Heart Association (AHA) resources, such as the AHA Workplace Wellness Kit and Fit-Friendly Workplaces resources | ❍ | ❍ | ❍ |
| Health Enhancement Research Organization (HERO) resources, such as the Employee Health Management (EHM) Best Practices Scorecard | ❍ | ❍ | ❍ |
| National Business Group on Health (NBGH) resources, such as the Wellness Impact Scorecard (WISCORE) | ❍ | ❍ | ❍ |
| WELCOA resources, such as the WELCOA Well Workplace Checklist | ❍ | ❍ | ❍ |
| Samueli Institute resources, such as the Samueli Institute Optimal Healthy Environments in the Workplace Assessment | ❍ | ❍ | ❍ |
| Partnership for Prevention resources, such as the Leading by Example resources | ❍ | ❍ | ❍ |
| Vitality Institute resources, such as workplace health related webinars and reports | ❍ | ❍ | ❍ |
| Chamber of Commerce resources | ❍ | ❍ | ❍ |

Please briefly describe any other helpful workplace health promotion resources (including meetings, trainings, and educational materials) that your Health Department has used in the past 12 months:

What resources (drawing from both the list provided two questions above, as well as any additional resources which you described) have been most helpful to your Health Department in performing workplace health promotion work? Please be as specific as possible, name up to three.

Resource 1: ______________________________

Resource 2: ______________________________

Resource 3: ______________________________

**Overall Commitment and Capacity**

Please rate your Health Department's commitment to workplace health promotion.

- ❍ Not at all committed
- ❍ Slightly committed
- ❍ Moderately committed
- ❍ Very committed
- ❍ Extremely committed

What level of priority does your Health Department assign to workplace health promotion efforts, in comparison to other efforts your Health Department is involved in?

- ❍ This is not a priority for my Health Department
- ❍ This is a low level priority for my Health Department
- ❍ This is a moderate level priority for my Health Department
- ❍ This is a high level priority for my Health Department
- ❍ This is a very high level priority for my Health Department

Please rate your Health Department's overall capacity to support workplace health promotion among employers in the state.

- ❍ No capacity
- ❍ Minimal capacity
- ❍ Some capacity
- ❍ Moderate capacity
- ❍ Substantial capacity

What specific types of assistance would your Health Department need to increase your capacity to support workplace health promotion? Consider aspects such as: human resources, financial resources, data resources, partnerships, and organizational culture, structure, and leadership:

A workplace health promotion program can help your Health Department support the health and well-being of your employees and serve as a model for other agencies and businesses in your state. Does your Health Department currently offer a wellness program to your employees?

- ❍ Yes
- ❍ No

**The following questions are about collaboration between Occupational Safety & Health staff and Workplace Health Promotion staff in your Health Department.**

Please rate your level of knowledge about occupational safety and health activities being performed by your Health Department.
- ❍ Know nothing
- ❍ Know some
- ❍ Know a moderate amount
- ❍ Know a substantial amount
- ❍ Extremely knowledgable

Do you ever collaborate with occupational safety and health staff in your Health Department on workplace safety and/or health promotion activities?
- ❍ Yes
- ❍ No

*If you answer **yes, you do collaborate**, you will see the following four questions:*

Please rate the level of collaboration between occupational safety and health and workplace health promotion staff in your Health Department on workplace safety and health promotion activities.
- ❍ Very low, we engage in limited collaborative projects and/or provide limited support to one another.
- ❍ Low
- ❍ Moderate, we work on some collaborative projects and/or provide some support to one another.
- ❍ High
- ❍ Very high, we work closely on collaborative projects and/or provide a great deal of support to one another.

Please describe one example of how you collaborate:

**Please rate your level of agreement with the following statements, from strongly disagree to strongly agree:**

Collaboration between occupational safety and health and workplace health promotion staff helps my Health Department achieve our aims for workplace safety and health promotion.

- ❍ Strongly disagree
- ❍ Somewhat disagree
- ❍ Neither agree nor disagree
- ❍ Somewhat agree
- ❍ Strongly agree

Collaboration between occupational safety and health and workplace health promotion staff in my Health Department is easy.

- ❍ Strongly disagree
- ❍ Somewhat disagree
- ❍ Neither agree nor disagree
- ❍ Somewhat agree
- ❍ Strongly agree

*If you answer* ***no, you do not collaborate****, you will see the following three questions:*

**Please rate your level of agreement with the following statements, from strongly disagree to strongly agree:**

Collaboration between occupational safety and health and workplace health promotion staff would help my Health Department achieve our aims for workplace safety and health promotion.

- ❍ Strongly disagree
- ❍ Somewhat disagree
- ❍ Neither agree nor disagree
- ❍ Somewhat agree
- ❍ Strongly agree

Collaboration between occupational safety and health and workplace health promotion staff in my Health Department would be easy.

❍ Strongly disagree
❍ Somewhat disagree
❍ Neither agree nor disagree
❍ Somewhat agree
❍ Strongly agree

Please describe the top three challenges to effective collaboration between occupational safety and health and workplace health promotion staff in your Health Department:

*All participants will see the following collaboration question:*
Do you collaborate with staff from any of the following public health programs in your Health Department?

| | Yes | No |
|---|---|---|
| Infectious disease program | ❍ | ❍ |
| Injury and violence prevention and control program | ❍ | ❍ |
| Cancer prevention and control program | ❍ | ❍ |
| Asthma program | ❍ | ❍ |
| Women's and children's health program | ❍ | ❍ |
| Other chronic disease prevention and control program(s), please describe: | ❍ | ❍ |
| Other types of programs in your Health Department, please describe:_________________ | ❍ | ❍ |

**The next few questions are about your Health Department's familiarity with "Total Worker Health".** Total Worker Health is a strategy supported by the Centers for Disease Control and Prevention's National Institute for Occupational Safety and Health (NIOSH) that integrates occupational safety and health protection with health promotion to prevent worker injury and illness and to advance worker health and well-being.

Please rate your level of familiarity with NIOSH's Total Worker Health initiative.
- ❍ Not familiar at all
- ❍ Slightly familiar
- ❍ Moderately familiar
- ❍ Very familiar
- ❍ Extremely familiar

**Please rate your level of agreement with the following statement, from strongly disagree to strongly agree:**
Applying an integrated approach to workplace safety and workplace health promotion is a priority for employers in my state.
- ❍ Strongly disagree
- ❍ Somewhat disagree
- ❍ Neither agree nor disagree
- ❍ Somewhat agree
- ❍ Strongly agree

Please indicate your Health Department's awareness and utilization of the following Total Worker Health-related resources:

| | Not aware | Aware, but has not utilized | Aware, and has utilized |
|---|---|---|---|
| The NIOSH Total Worker Health webinar series | ❍ | ❍ | ❍ |
| The NIOSH Total Worker Health in Action! eNewsletter | ❍ | ❍ | ❍ |
| The "SafeWell Practice Guidelines: an Integrated Approach to Worker Health v2.0" from the Harvard School of Public Health's Center for Work, Health, & Well-Being | ❍ | ❍ | ❍ |
| The "Healthy Workplace Participatory Program" online toolkit from the Center for the Promotion of Health in the New England Workplace | ❍ | ❍ | ❍ |
| "The Whole Worker: Guidelines for Integrating Occupational Health and Safety with Workplace Wellness Programs" from the State of California's Commission on Health and Safety and Workers' Compensation | ❍ | ❍ | ❍ |
| The "Indicators of Integration" assessment tool from the Harvard School of Public Health's Center for Work, Health, & Well-Being | ❍ | ❍ | ❍ |
| The "Corporate Health Achievement Award self-assessment" from the American College of Occupational and Environmental Medicine | ❍ | ❍ | ❍ |
| The Journal of Occupational and Environmental Medicine's Total Worker Health Supplement (Vol 55, Supplement 12S, Dec 2013) | ❍ | ❍ | ❍ |
| The "CPH News & Views" bi-monthly emerging topics briefs from the Center for the Promotion of Health in the New England Workplace | ❍ | ❍ | ❍ |
| The "Total Worker Health Essentials" video series from the University of Iowa's Healthier Workforce Center for Excellence | ❍ | ❍ | ❍ |

What resources (drawing from both the list provided above, as well as any additional resources which you are aware of) have been most helpful to your Health Department in performing integrated workplace safety and health promotion work? Please be as specific as possible, name up to three.

Resource 1: ______________________________

Resource 2: ______________________________

Resource 3: ______________________________

**We would now like to ask some questions about how workplace health promotion is seen by different stakeholders in your state.** Please rate your level of agreement with the following statements, from strongly disagree to strongly agree.

Improving workers' health behaviors and overall health & well-being is a priority for employers in my state.

- ❍ Strongly disagree
- ❍ Somewhat disagree
- ❍ Neither agree nor disagree
- ❍ Somewhat agree
- ❍ Strongly agree

Improving workers' health behaviors and overall health & well-being is a priority for labor unions in my state.

- ❍ Strongly disagree
- ❍ Somewhat disagree
- ❍ Neither agree nor disagree
- ❍ Somewhat agree
- ❍ Strongly agree

Improving workers' health behaviors and overall health & well-being is a priority for legislators in my state.

- ❍ Strongly disagree
- ❍ Somewhat disagree
- ❍ Neither agree nor disagree
- ❍ Somewhat agree
- ❍ Strongly agree

Improving workers' health behaviors and overall health & well-being is a priority for the governor in my state.
- ❍ Strongly disagree
- ❍ Somewhat disagree
- ❍ Neither agree nor disagree
- ❍ Somewhat agree
- ❍ Strongly agree

**The following questions are about the Affordable Care Act and worker health in your state.**
In this section, smaller workplaces are defined as those with less than 250 employees.

How has the Affordable Care Act impacted the number of workplaces which are offering workplace health-promotion programs? Do you think the number of workplaces has...
- ❍ Greatly decreased
- ❍ Somewhat decreased
- ❍ Neither decreased nor increased
- ❍ Somewhat increased
- ❍ Greatly increased

How has the Affordable Care Act impacted the number of **smaller** workplaces which are offering workplace health-promotion programs? Do you think the number of workplaces has...
- ❍ Greatly decreased
- ❍ Somewhat decreased
- ❍ Neither decreased nor increased
- ❍ Somewhat increased
- ❍ Greatly increased

How has the Affordable Care Act impacted the number of workplaces which are offering employees financial incentives to participate in health-contingent wellness programs linked to health insurance. Do you think the number of workplaces has...
- ❍ Greatly decreased
- ❍ Somewhat decreased
- ❍ Neither decreased nor increased
- ❍ Somewhat increased
- ❍ Greatly increased

How has the Affordable Care Act impacted the number of **smaller** workplaces which are offering employees financial incentives to participate in health-contingent wellness programs linked to health insurance. Do you think the number of workplaces has...

- ❍ Greatly decreased
- ❍ Somewhat decreased
- ❍ Neither decreased nor increased
- ❍ Somewhat increased
- ❍ Greatly increased

**Interviews**

Over the summer, the research team at the University of North Carolina Gillings School of Global Public Health will be conducting interviews with selected State Health Departments to gain a deeper understanding of workplace safety and health promotion activities and capacity. Do we have your permission to contact you about participating in a follow-up interview?

- ❍ Yes
- ❍ No
