# Supplementary material for: Workplace health promotion and safety in state and territorial health departments in the United States: a national mixed-methods study of activity, capacity, and growth opportunities
Source: BMC Public Health. 2019 Mar 12;19:291. doi: 10.1186/s12889-019-6575-x (PMC6417036; doi:10.1186/s12889-019-6575-x)
Supplement: Supplementary file 3 — National Survey of State and Territorial Health Departments’ Workplace Health and Safety Activities: Follow-up Interview Questions (OSH version) (PDF 179 kb) [file 12889_2019_6575_MOESM3_ESM.pdf]

National Survey of State and Territorial Health Departments' Workplace Health and Safety Activities: Follow-up Interview Questions (OSH version)

Thank you for making the time for this interview. As we mentioned before, the overall goal of this interview is to get your thoughts on what the CDC could do to best work with states in the field of worker health and safety. We also want to explore in more depth some topics that you and others told us about in the survey. The interview will last 45-60 minutes, but, all questions are voluntary and if there are questions you prefer not to answer, just let me know and we will move to the next.

IQ1. To begin, I'd like to ask a little more about you in your current job. What percent of your time would you say you spend doing OSH*-related tasks?

IQ2. Can you tell me a little about what kinds of OSH-related tasks you are responsible for?

(Ask the next question if two participants are confirmed for the interview)

IQ3. [Original survey respondent], throughout the remainder of the interview, we would like to ask some follow-up questions about some of your survey responses. This means we would be discussing the responses which you gave in the survey with you and [co-worker/invited participant]. If that is NOT OK, please let me know now. We can still go through some questions which are not based on your survey responses.

- ***(if participant does NOT want to discuss survey responses with co-worker present, only ask IQs 8, 13-19, 21, 23-24, 27, 29-31, 33-36, 38 – those which do not draw on survey responses)***
- ***(if participant does want to discuss survey responses with co-worker present, CONTINUE with next question)***

One of the topics that we want to explore in more depth are the partnerships you might have with people or organizations **outside of your health department**….

(*Ask the next question if, in response to Q11.13, the respondent reported a partnership for one of the four implementation support activities*)

IQ4. In the survey, you said that during the past year, your health department worked with partners in order to [populate with response to Q11.13: provide educational materials for employers/train employers/provide technical assistance to employers/help employers monitor the quality of their workplace health programs]. Can you tell me more about an example of one of these partnerships?

- **[INSERT PARTNERSHIP PROBES[i]]**

(*Ask the next question if, in response to Q11.13, the respondent did NOT report a partnership for one of the four implementation support activities*)

IQ5. In the survey, you said that during the past year, your health department did NOT work with partners in order to provide educational materials for employers, train employers, provide technical assistance to employers, or help employers monitor the quality of their workplace health programs. Is this still true?"

- **(*if there IS a partnership for one of these four activities*)** Can you tell me more about an example of one of these partnerships? *(go to **PARTNERSHIP PROBES[i]**)*
- ***(if still NO partnership for these four activities, go to next question)***

(Ask this question if NO implementation support partnerships in Q11.13, YES surveillance partnerships Q11.2)

IQ6. In the survey, you said that during the past year, your health department worked with partners in order to conduct OSH surveillance activities. Can you tell me more about an example of one of these partnerships?

- **[INSERT PARTNERSHIP PROBES[i]]**

(Ask this question if NO implementation support partnerships in Q11.13, NO surveillance partnerships Q11.2, YES direct service partnerships Q11.42)

IQ7. In the survey, you said that during the past year, your health department worked with partners in order to deliver OSH services directly to workers. Can you tell me more about an example of one of these partnerships?

- **[INSERT PARTNERSHIP PROBES[i]]**

(CONTINUE interview w/ question below)

IQ8. What advice would you give to other state health departments about forming partnerships to address workplace health and safety?

Thank you. One of the next topics we are really interested to learn more about from Health Departments is the relationship between staff doing OSH projects and staff doing WHP[1] projects in your department.

(*Ask the next question if, in response to Q14.3, the respondent said that they collaborated with WHP staff and in Q14.5 gave an example of how they collaborated*)

IQ9. In the survey, you said that you collaborated with WHP staff in your health department to [populate with response to Q14.5]. Can you tell me a little more about this collaboration?

- [**INSERT COLLABORATION PROBES**[ii]]

(Ask the next question if, in response to Q14.3, the respondent said that they collaborated with WHP staff but in Q14.5 gave NO example of how they collaborated)

IQ10. In the survey, you said that you collaborated with WHP staff in your health department. Could you tell me about an example of a project that you did with them?

- [**INSERT COLLABORATION PROBES[ii]**]

[1] In this study, **workplace health promotion**, or **workplace wellness**, is defined as the use of workplace programs, policies, and environmental supports to support healthy behaviors and improve employee health.

(Ask the next question if, in response to Q14.3, the respondent said that they did NOT collaborate with WHP staff)

IQ11. In the survey, you indicated that there were no collaborations between WHP staff and OSH staff in your health department. Is that statement still true?

- ***(if there IS a new collaboration)*** Can you tell me a little more about this collaboration? *(go to **COLLABORATION PROBES**[ii])*
- ***(if still NO collaboration)*** Are there any WHP staff in your Health Department currently?
- ***(if still NO collaboration, irrespective of answer to above probe)*** While you've been working at your department, has there ever been a time when there was collaboration between staff doing WHP work and staff doing OSH work?
    1. ***(if YES collaboration, go to COLLABORATION PROBES**[ii]**)***
    2. ***(if NO collaboration)*** While you've been working at your department, have you ever been involved in any collaborations with WHP staff at other organizations?
        a. ***(if YES collaboration, go to COLLABORATION PROBES**[ii]**)***
        b. ***(if NO collaboration, continue with interview, starting with next question)***

(***CONTINUE interview w/ question below***)

(Ask this next question if only one participant is confirmed for the interview)

IQ12. In the survey, we asked you how much you agreed or disagreed with the following statement: "Collaboration between OSH staff and WHP staff [if respondent answered Q14.7: "helps"/if respondent answered Q14.9: "would help"] my health department achieve our aims for workplace safety and health promotion." You said that you [populate with response to Q14.7 or

Q14.9]. Can you tell me more about what made you [agree/neutral/disagree]?

- Is there anything that could happen that would change your rating from [populate with response to Q14.7 or Q14.9]?

(Ask this next question if two participants are confirmed for the interview)

IQ13. I'd like to ask you each the following question independently.

- [First respondent], Please rate your level of agreement with the following statement: "Collaboration between occupational safety and health and workplace health promotion staff helps my Health Department achieve our aims for workplace safety and health promotion". Would you say that you Strongly disagree, Somewhat disagree, Neither agree nor disagree, Somewhat agree, or Strongly agree? (***note 1st respondent's answer***)
- [Second respondent], Please rate your level of agreement with the following statement: "Collaboration between occupational safety and health and workplace health promotion staff helps my health Department achieve our aims for workplace safety and health promotion". Would you say that you Strongly disagree, Somewhat disagree, Neither agree nor disagree, Somewhat agree, or Strongly agree? (***note 2nd respondent's answer, then probe both respondents with the following probes***)
- Can you tell me more about what made you [agree/neutral/disagree]?
- Is there anything that could happen that would change your rating from [response]?

(CONTINUE interview w/ question below)

IQ14. What do you think would need to happen for there to be greater collaboration between WHP and OSH staff?

This discussion has been really helpful to giving me a better picture of the kind of work your health department does, and who you work with. I'd like to switch the focus of the

interview a little, towards what your health department may need to expand efforts in workplace health, well-being, and safety…

IQ15. What kinds of projects or services would be highest priority if your department were able to expand its efforts in occupational safety and health?

IQ16. What do you think your department would need the most right now if it wanted to expand its efforts in occupational safety and health?

- (***The participant may focus on staff and funding in response to this question. If so, use this probe***) Now please set aside the idea of getting more staff or funding. What else might do the most to help you expand occupational safety and health?

Now I'd like to ask about some specific follow-up questions about resources in your division.

First, I'd like to start with funding. I know that funding can be complicated to describe, but, generally speaking, how…

IQ17. …are OSH surveillance activities funded in this state, if you do them?

IQ18. …is OSH work that you do with employers funded in this state, if you do that?

IQ19. …is OSH work that you do directly with workers funded in this state, if you do work with workers?

(*Ask the next question if, in response to Q13.6, respondent gave a funding estimate)*

IQ20. This next question is to help us understand our data better. In the survey, you responded that your state's total funding for OSH activities over the past 12 months was [populate with response to Q13.6]. How did you arrive at this number? (***use the probes below as needed to clarify***)

- Did you include the salary of staff doing OSH work in this estimate?
- Did you include any funds which are dedicated to providing OSH services for SHD employees?

- (***if respondent wants to provide updated estimate, ensure that this DOES include salary, but does NOT include funds dedicated to providing SHD employees with OSH services***)

(CONTINUE interview w/ question below)

IQ21. What do you think is important for us to know when it comes to funding for occupational safety and health programming?

Now I would like to ask a few questions about staff, and staff training…

IQ22. In the survey, you reported that there were [populate with response to Q13.3] FTEs in your department doing OHS activities.

- (*if Q13.3=>0, include this probe*) What types of staff, in terms of role, were included in this estimate? (***use 1-3 below as needed to clarify***)
  1. Were any of these staff responsible for providing OSH services to non-SHD workers and employers across the state?
  2. Were any of these staff responsible for OSH surveillance?
  3. Were any of these staff responsible for providing OSH services to SHD employees?
- Are there any staff doing OSH-related activities which you excluded from this estimate? (***if yes***) What types of OSH-related work do they do?
- Our intent in asking about the number of FTEs was get an estimate which was adjusted to reflect the % of full-time that each person contributed to OSH activities, excluding any OSH services provided to SHD employees. In light of this, would you like to make any changes to the estimate which you gave?

IQ23. In your opinion, what might your Health Department's ideal staffing be to do OSH work in this state?

IQ24. When hiring staff to do OSH work, what kinds of expertise and/or training do you expect staff to have?

(Ask the next question if, in response to Q13.10, the respondent gave an answer which focused on knowledge or skills)

IQ25. In the survey, you mentioned that you thought it would be good for staff in your health department to have more knowledge about [populate with survey response to Q13.10]. Can you think of any trainings or resources that the CDC could offer to help meet these needs? (***If yes***) Please describe.

(Ask the next question if, in response to Q13.10, the respondent gave an answer which did NOT focus on knowledge or skills, or gave no answer)

IQ26. Can you think of any trainings or resources that the CDC could offer to help staff in your department improve their competency to perform occupational safety and health activities? (***If yes***) Please describe.

IQ27. Can you think of any trainings that have been particularly helpful to you in your work as an OSH practitioner? ***(if yes, probe on each training mentioned)*** What characteristics of that training made it so helpful?

Finally, I'd like to ask about leadership and support you receive for occupational safety and health in this state…

(Ask this next question if only one participant confirmed for the interview)

IQ28. In the survey, you described your health department as being [populate with response to Q13.16] committed to OSH. Could you explain why you characterized "commitment" in that way?

(Ask this next question if two participants are confirmed for the interview)

IQ29. I'd like to ask you each the following question independently.

- [First respondent], Please rate your Health Department's commitment to occupational safety and health. Would you say your health department is: Not at all committed, Slightly committed, Moderately committed, Very committed, or Extremely committed? (***note 1st respondent's answer***)

- [Second respondent], Please rate your Health Department's commitment to occupational safety and health. Would you say your health department is: Not at all committed, Slightly committed, Moderately committed, Very committed, or Extremely committed?" (***note 2nd respondent's answer, then probe both respondents with the following probe***)
    - Could you explain why you characterized "commitment" in that way?

(CONTINUE interview w/ question below)

IQ30. What could your health department leadership do to better support OSH in your state?

IQ31. Can you think of any ways the CDC could help Health Department leadership become more supportive of OSH? ***(if yes)*** Please explain.

I have one more follow-up question about resources…

IQ32. We asked about helpful resources that your health department uses to conduct OSH work. You replied that [populate with responses to Q13.14, if no answer remove question] were the most helpful resources. Of those three, pick the one you consider to be the most helpful…

- In what way did you use [the most helpful resource]?
- What characteristics made it so helpful?
- Is there anything you would change?

To help give us some perspective on your work, I'd like to ask a few questions about how you see your health department's role in the field of workplace safety, health and well-being, overall…

IQ33. Considering all of the different organizations that focus on worker health and safety in your state, what role does your health department play in protecting and promoting workers' health?

- How does this compare with the role of Other State Agencies which do OSH work?

IQ34. What would you say are the most important OSH programs or services that your Health Department offers?

- How does this compare with the role of Other State Agencies which do OSH work?

IQ35. What would you say are the most important OSH issues in your state right now?

IQ36. What types of employers does your department currently work with on OSH? Is there a certain type of employer by industry sector or size that you prioritize?

I have two closing questions. First, now that we have talked in-depth about the work that your health department does, I'd like to review with you how we coded your state's activity level in comparison with other states…

IQ37. When we reviewed survey responses, our team coded the level of OSH activity for each respondent as none, low, or high. We coded your health department as [none; low; high] on OSH activities. Do you agree with this rating? Why or why not?

IQ38. Is there anything else you want the CDC to know about in order to build the capacity of state health departments to offer workplace health and safety services?

---

[i] **PARTNERSHIP PROBES**

- How did the partnership start?
- What role did your health department play in this partnership? What role(s) did the partner(s) play?
- What motivated your department to work with a partner?

- What has been positive about working with a partner? (***if focusing on project outcomes***) What is it about this *partnership* that made the outcome positive?
- What challenges did you have to overcome in this partnership?
- Would [you/your Health Department] engage in a partnership like this again? Why or why not?

[ii]**COLLABORATION PROBES**

- How did the collaboration start?
- What role did WHP staff play in this collaboration? What role did [you/OSH staff] play?
- What made OSH and WHP staff decide to work in collaboration?
- What do you believe were the positive outcomes for your work, if any?
- If you were to guess, what do you believe were the positive outcomes for your WHP collaborators, if any?
- What were the challenges associated with this collaboration, if any?
    1. What could have addressed these challenges?
- If you were to guess, what do you believe were the challenges for your WHP collaborators, if any?
- Would [you/your Health Department] engage in a collaboration like this again? Why or why not?
