# Supplementary material for: Workplace health promotion and safety in state and territorial health departments in the United States: a national mixed-methods study of activity, capacity, and growth opportunities
Source: BMC Public Health. 2019 Mar 12;19:291. doi: 10.1186/s12889-019-6575-x (PMC6417036; doi:10.1186/s12889-019-6575-x)
Supplement: Supplementary file 4 — Occupational Safety and Health (OSH) Surveillance Activities. The prevalence of specific OSH surveillance activities as reported by survey respondents. (PDF 100 kb) [file 12889_2019_6575_MOESM4_ESM.pdf]

**National Survey of State and Territorial Health Departments' Workplace Health and Safety Activities: Occupational Safety and Health (OSH) Surveillance Activities**		
		**OSH survey respondents (n=39)**
SHD compiles, analyzes, and interprets Occupational Health Indicators (OHIs)		72% (28)
How many OHIs are tracked?		
0-14	8% (3)	
15-21	38% (15)	
All 22	26% (10)	
**SHD (independently, in partnership, or through a bona fide agent) conducts…**		
…surveillance of occupational lead levels in adults and submission of data to the NIOSH Adult Blood Lead Epidemiology and Surveillance (ABLES) program		85% (33)
…Workers' Compensation surveillance, beyond the collection of Workers' Compensation-related OHIs		49% (19)
…targeted surveillance of target worker populations		49% (18)
…monitoring of indicators for the Healthy People 2020 occupational safety and health (OSH) objectives		38% (14)
…targeted surveillance for fatality assessment, control, and evaluation (FACE)		37% (14)
…targeted surveillance of pesticide illness and injury		36% (13)
…targeted surveillance of occupational respiratory disease		35% (13)
…monitoring of data from the National Surveillance System for Pneumoconiosis Mortality (NSSPM)		19% (7)
…targeted surveillance of musculoskeletal disorders		19% (7)
**SHD (independently, in partnership, or through a bona fide agent)…**		
…presents OSH surveillance data to relevant staff (occupational health professionals and health care providers) at workplaces within their state		62% (24)
