# Supplementary material for: Workplace health promotion and safety in state and territorial health departments in the United States: a national mixed-methods study of activity, capacity, and growth opportunities
Source: BMC Public Health. 2019 Mar 12;19:291. doi: 10.1186/s12889-019-6575-x (PMC6417036; doi:10.1186/s12889-019-6575-x)
Supplement: Supplementary file 5 — Implementation Supports – How Many Employers Are Reached?. Survey respondents’ report of how many employers receive OSH/WHP tools, trainings, technical assistance, and/or quality assurance/quality improvement from the health department. (PDF 98 kb) [file 12889_2019_6575_MOESM5_ESM.pdf]

**National Survey of State and Territorial Health Departments' Workplace Health and Safety Activities: Implementation Supports – How Many Employers Are Reached?**						
	SHD does not provide this service	SHD does not track employers reached	0-25 employers	26-50 employers	51-100 employers	101 or more employers
**How many employers are reached by each type of OSH implementation support?**						
Tools	32% (12)	32% (12)	11% (4)	3% (1)	5% (2)	16% (6)
Training	61% (22)	17% (6)	8% (3)	3% (1)	8% (3)	3% (1)
Technical Assistance	39% (14)	17% (6)	42% (15)	3% (1)	0% (0)	0% (0)
Quality Assurance/Improvement	82% (28)	6% (2)	6% (2)	3% (1)	0% (0)	3% (1)
**How many employers are reached by each type of WHP implementation support?**						
Tools	10% (4)	15% (6)	10% (4)	8% (3)	15% (6)	43% (17)
Training	23% (9)	8% (3)	13% (5)	10% (4)	20% (8)	28% (11)
Technical Assistance	21% (8)	8% (3)	18% (7)	13% (5)	16% (6)	24% (9)
Quality Assurance/Improvement	38% (14)	14% (5)	8% (3)	11% (4)	14% (5)	16% (6)

*n=39 OSH respondents and n=40 WHP respondents
